# Revealing the Mechanism of *Hemerocallis citrina* Baroni in Depression Treatment Through Integrated Network Pharmacology and Transcriptomic Analysis

**DOI:** 10.3390/ph17121704

**Published:** 2024-12-17

**Authors:** Shan Gao, Jihui Lu, Yixiao Gu, Yaozhi Zhang, Cheng Wang, Feng Gao, Ziqi Dai, Shujing Xu, Jindong Zhang, Yuqin Yang, Haimin Lei

**Affiliations:** 1 School of Chinese Pharmacy, Beijing University of Chinese Medicine, Beijing 102488, China; 18813009163@163.com (S.G.); ljh980614@163.com (J.L.); 20220931101@bucm.edu.cn (Y.G.); zhangyaozhi1111@163.com (Y.Z.); chengwangcr@163.com (C.W.); gaofeng_1996@126.com (F.G.); 15735646823@163.com (Z.D.); xusj0318@163.com (S.X.); 18843599361@163.com (J.Z.); 2 Institute of Medicinal Plant Development, Chinese Academy of Medical Sciences and Peking Union Medical College, Beijing 100193, China; 3 Institute of Basic Theory for Chinese Medicine, China Academy of Chinese Medical Sciences, Beijing 100700, China

**Keywords:** *Hemerocallis citrina* Baroni, depression, network pharmacology, transcriptomic analysis, PI3K/Akt/CREB signaling pathway

## Abstract

**Background/Objectives**: *Hemerocallis citrina* Baroni (HCB) is a traditional herb for the treatment of depression in China. However, the active constituents and the underlying mechanisms of its antidepressant effects remain unclear. The aim of this study was to identify the bioactive constituents of HCB and elucidate its underlying mechanism for the treatment of depression. **Methods**: The constituents of HCB were systematically analyzed using UHPLC-Q-Orbitrap HRMS. Its antidepressant effect was evaluated by chronic unpredictable mild stress (CUMS)-induced depression. The mechanism of HCB in treating depression was investigated through network pharmacology and molecular docking. Subsequently, its potential mechanism for the treatment of depression was carried out by RNA sequencing. Finally, the mechanism was further verified by Western blot. **Results**: A total of 62 chemical constituents were identified from HCB using UHPLC-Q-Orbitrap HRMS, including 17 flavonoids, 11 anthraquinones, 11 alkaloids, 10 caffeoylquinic acid derivatives, five phenolic acids, five triterpenoids, and three phenylethanosides, 13 of which were identified as potential active constituents targeting 49 depression-associated proteins. Furthermore, HCB was found to significantly reduce cognitive impairment, anxiety-like behavior, and anhedonia-like behavior. The expression levels of 5-hydroxytryptamine (5-HT), dopamine (DA), and brain-derived neurotrophic factor (BDNF) were elevated in the hippocampal CA3 region. Results from network pharmacology and transcriptomics indicated that the PI3K/Akt/CREB signaling pathway is essential for the therapeutic effects of HCB on depression. Research in the field of molecular biology has conclusively demonstrated that HCB is associated with an increase in the expression levels of several important proteins. Specifically, there was a notable upregulation of phosphorylated PI3K (p-PI3K) relative to its unphosphorylated form PI3K, as well as an elevation in the ratio of phosphorylated Akt (p-Akt) to total Akt. Additionally, the study observed increased levels of phosphorylated CREB (p-CREB) compared to its unphosphorylated CREB. **Conclusions**: This study provides compelling evidence that HCB possesses the ability to mitigate the symptoms of depression through its influence on the PI3K/Akt/CREB signaling pathway. HCB could be developed as a promising therapeutic intervention for individuals struggling with depression, offering new avenues for treatment strategies that target this particular signaling mechanism.

## 1. Introduction

Depression is a psychiatric illness characterized by cognitive decline and a prolonged gloomy mood, which significantly reduces quality of life [1,2]. In today’s fast-paced world, people are more prone to experiencing psychological issues, such as anxiety and depression, due to work pressures and the overall pace of life. According to the World Health Organization (WHO), depression is expected to affect 350 million people globally by 2030, becoming the leading cause of disease burden [3]. This highlights the immense economic burden of depression and underscores the need for greater efforts to improve treatment, especially in low-income and middle-income countries with limited healthcare resources [4].

Currently, clinically used antidepressants have several drawbacks, including lengthy treatment times and physical discomfort. Additionally, drug dependence can hinder optimal therapeutic outcomes and may lead to drug resistance and toxic side effects. For instance, the prolonged use of fluoxetine can cause insomnia, headaches, and diarrhea [5]. As a result, seeking complementary therapies to traditional medicines is a wise approach.

With recent societal developments, plant-based resources that have both medicinal and dietary uses have gained attention as botanical additives for the prevention and treatment of various diseases, owing to their low toxicity and minimal side effects [6]. *Hemerocallis citrina* Baroni (HCB), often referred to as the daylily, belongs to the Hemerocallis family of plants [7,8]. Historical medical texts, such as Ben Cao Gang Mu, mention the use of HCB for reducing swelling, stopping bleeding, relieving lumbago, treating jaundice, and addressing breast carbuncles and sores [9].

In recent years, some studies have reported on the antidepressant effects of HCB in both clinical and experimental settings. These studies suggest that the ethanol extract of HCB has antidepressant effects by regulating neurotransmitter metabolism in specific brain regions [10]. Additionally, ethanol extract from HCB has been shown to upregulate BDNF and TrkB receptor proteins in the hippocampus of rats, alleviating depression-like behaviors [11]. However, the specific pharmacodynamic substances and mechanisms of HCB in treating depression remain unclear, limiting its broader application. Thus, there is an urgent need to clarify the active constituents of HCB and its underlying mechanisms in treating depression.

In this study, the chemical constituents of HCB were identified using UHPLC-Q-Orbitrap HRMS [12], which is the basis for determining its antidepressant activity. The antidepressant effects were evaluated using the chronic unpredictable mild stress (CUMS) model. Subsequently, the complex mechanisms of HCB in treating depression were revealed by integrating network pharmacology with transcriptomics analysis [13]. Finally, HCB achieves antidepressant effects through the PI3K/Akt/CREB signaling pathway, which was further validated by Western blot. Above all, this study demonstrates that HCB could be a potent complementary medicine for the treatment of depression.

## 2. Results

### 2.1. Identification of the Constituents in HCB

**Figure 1:**
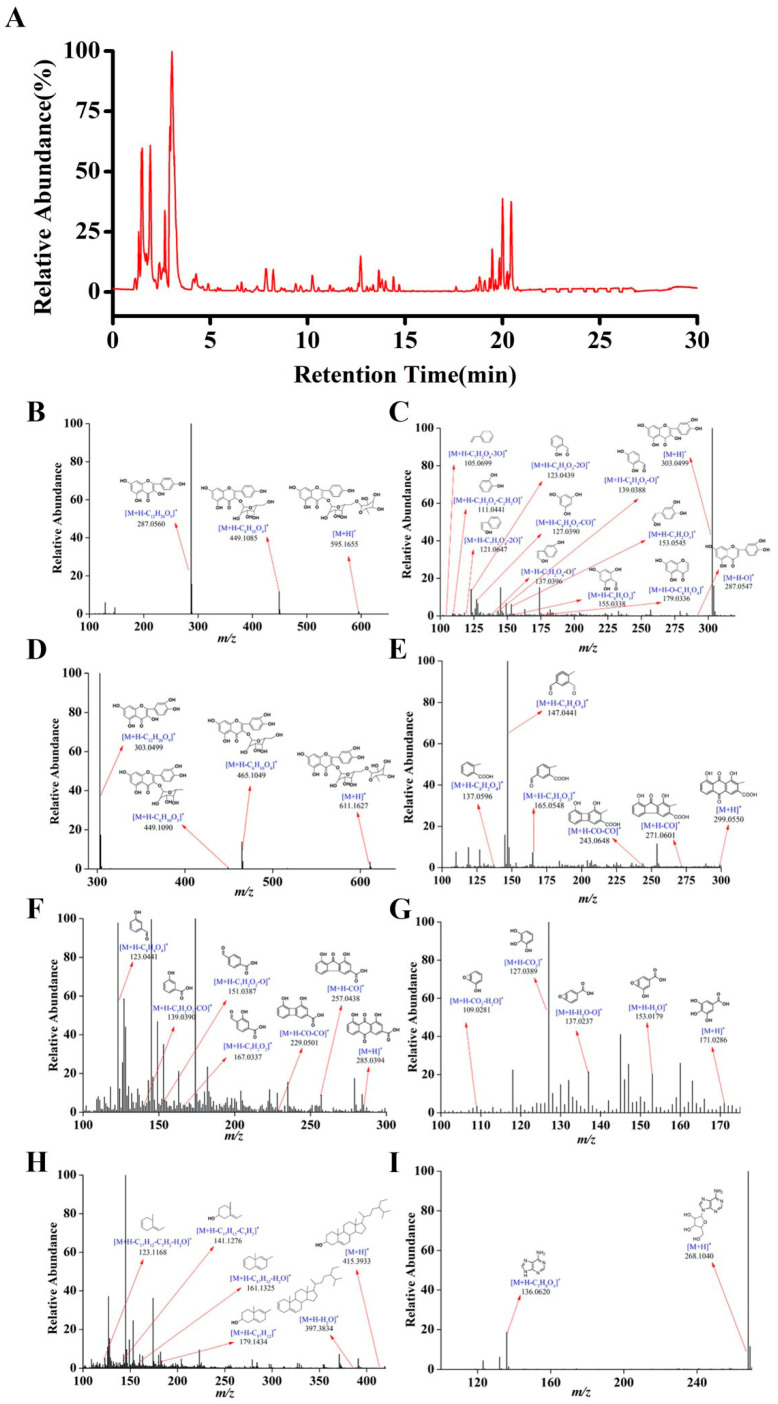
Mass spectrogram of HCB. (**A**) TICC of HCB obtained in ESI+ mode. Product ion spectra of (**B**) kaempferol-3-rutinoside. (**C**) quercetin. (**D**) rutin. (**E**) kwanzoquinone G. (**F**) rhein. (**G**) gallic acid. (**H**) clionasterol. (**I**) adenosine.

To comprehensively evaluate the chemical constituents of the ethanol extract of HCB, the bioactive constitutes of the ethanol extract of HCB were determined by UHPLC-Q-Orbitrap-HRMS. The total ion current chromatogram (TICC) of the ethanol extract HCB in the ESI^+^ mode was shown in Figure 1A. In general, 62 chemical constituents, including 17 flavonoids, 11 anthraquinones, 11 alkaloids, 10 caffeoylquinic acid derivatives, five phenolic acids, five triterpene, and three phenylethanoid glycosides were identified from the ethanol extract HCB. The mass spectrometric data are listed in Table 1. A protonated molecule of kaempferol-3-rutinoside was detected at *m*/*z* 595.1655 in positive ionization mode as [M+H]^+^. This protonated species subsequently lost a C_6_H_10_O_4_ group, resulting in a fragment ion [M+H-C_6_H_10_O_4_]^+^ with a mass-to-charge ratio of *m*/*z* 449.1085. Further fragmentation occurred, as it lost a C_6_H_10_O_5_ moiety, producing another fragment ion [M+H-C_12_H_20_O_9_]^+^ at *m*/*z* 259.06 (Figure 1B, Table 1). Quercetin existed in the form of [M+H]^+^ at *m*/*z* of 303.0499 in positive ionization mode (Figure 1C, Table 1). The molecule that was protonated experienced the loss of an O moiety from the C-ring, resulting in the formation of a fragment ion [M+H-O]^+^ observed at *m*/*z* 287.0547. Subsequent losses of C_6_H_4_O_4_ led to the detection of product ions [M+H-O-C_6_H_4_O_4_]^+^ at *m*/*z* 179.0336. Additionally, the fragment ion detected at *m*/*z* 153.0545 ([M+H-C_7_H_2_O4]^+^) was a result of losing C_7_H_2_O_4_. Due to further losses of O and C_2_H_2_O, product ions [M+H-C_7_H_2_O_4_-O]^+^ at *m*/*z* 137.0396 and [M+H-C_7_H_2_O_4_-C_2_H_2_O]^+^ at *m*/*z* 111.0441 were observed. Rutin exhibited a protonated molecule [M+H]^+^ at *m*/*z* 611.1627 in positive ionization mode. A characteristic fragment ion, [M+H-C_6_H_10_O_4_]^+^ at *m*/*z* 465.1049, was produced by the loss of one C_6_H_10_O_4_ molecule from *m*/*z* 611.1627, which formed the fragment ions [M+H-C_12_H_20_O_9_]^+^ at *m*/*z* 303.0499 and [M+H-C_6_H_10_O_9_]^+^ at *m*/*z* 449.1090 as a result of further losses of C_6_H_10_O_5_ and O molecules (Figure 1D, Table 1). The molecular formula of kwanzoquinone G was C_16_H_10_O_6_, which indicated the detection of a protonated molecule [M+H]^+^ at *m*/*z* 299.0500 in the positive ion mode (Figure 1E, Table 1). Rhein produced a protonated molecule [M+H]^+^ at *m*/*z* 285.0394 under the same ionization conditions. Subsequently, this protonated form underwent the loss of a CO fragment, resulting in the ion [M+H-CO]^+^ at *m*/*z* 257.0438, which further experienced the loss of another CO to form the ion [M+H-2CO]^+^ at *m*/*z* 229.0501 (Figure 1F, Table 1). Gallic acid was found to generate a protonated molecule [M+H]^+^ at *m*/*z* 171.0286 in positive ion mode. This protonated entity depleted a CO_2_ unit, yielding a fragment ion [M+H-CO_2_]^+^ at *m*/*z* 127.0389, which then lost H_2_O to produce the ion [M+H-CO_2_-H_2_O]^+^ at *m*/*z* 109.0281 (Figure 1G, Table 1). In the case of Clionasterol, a protonated species [M+H]^+^ at *m*/*z* 415.3933 was noted in the positive ion setting. Fragmentations at *m*/*z* 179.1714 emerged as a result of RDA fragmentation. Further losses of C_3_H_2_ and H_2_O generated product ions, such as [M+H-C_17_H_12_-C_3_H_2_]^+^ at *m*/*z* 141.1276, [M+H-C_17_H_12_-C_3_H_2_-H_2_O]^+^ at *m*/*z* 123.1168, and [M+H-C_17_H_12_-H_2_O]^+^ at *m*/*z* 179.1434 (Figure 1H). The molecular formula of adenosine was C_10_H_13_N_5_O_4_, and adenosine exhibited a protonated molecule [M+H]^+^ at *m*/*z* 268.1040 in positive ionization mode (Figure 1H,I, Table 1, Figures S1–S11).

### 2.2. HCB Attenuates CUMS-Induced Depression

**Figure 2:**
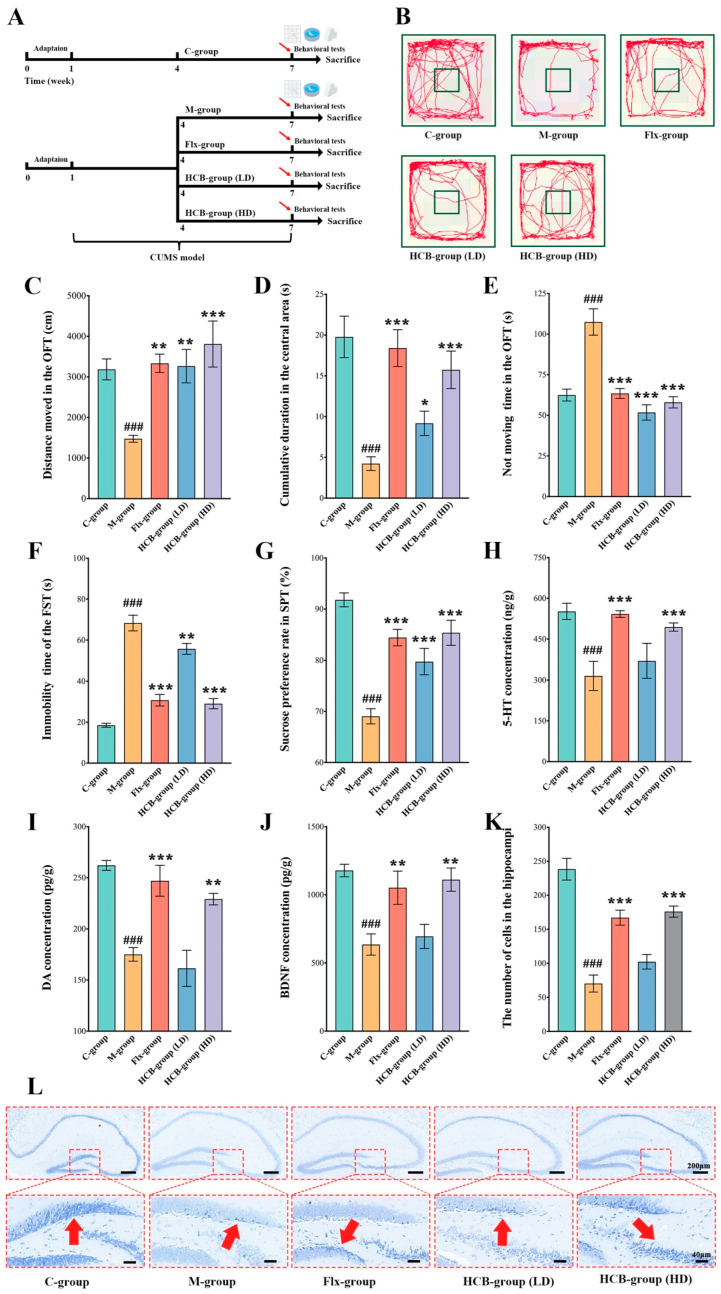
HCB improved CUMS mice depression-like behaviors. (**A**) Schematic diagram of experimental design. (**B**) Representative images of movement trajectory. (**C**) Total distance within 5 min in the OFT (*n* = 8). (**D**) Time spent in the central area in the OFT (*n* = 8). (**E**) Not moving time in the OFT within 5 min (*n* = 8). (**F**) Immobility time in the FST within 4 min (*n* = 8). (**G**) Changes in precent of sucrose preference in the SPT (*n* = 8). (**H**) The secretion levels of 5-hydroxytryptamine (*n* = 3). (**I**) The secretion levels of dopamine (*n* = 3). (**J**) The secretion levels of BDNF (*n* = 3). (**K**) The number of Nissl bodies in the hippocampal CA3 regions (*n* = 3). (**L**) Representative pictures of Nissl staining in the hippocampi. Data are presented as mean ± SEM, ^###^ *p* < 0.001 vs. control group (C-group); * *p* < 0.05, ** *p* < 0.01, *** *p* < 0.001 vs. model group (M-group).

To assess the efficacy of HCB on depression, a chronic unpredictable mild stress (CUMS)-induced depression model was established in mice. The experimental design is illustrated in Figure 2A. As shown in Figure 2B,C, compared to the control group (C-group), the total distance traveled and the immobility time within 5 min in the central area of the model group (M-group) were significantly reduced during the open field test (OFT) (*p* < 0.001), indicating decreased activity and cognitive ability in the M-group (Figure 2D). The total distance traveled by the HCB low-dose group (HCB-group LD) and high-dose group (HCB-group HD) was significantly greater than that of the M-group (*p* < 0.01, *p* < 0.001). Furthermore, the HCB-group HD spent more time in the central area compared to the M-group (*p* < 0.001). The immobility time in the HCB-group LD and HCB-group HD was significantly decreased compared to the M-group (*p* < 0.001 for both) (Figure 2E).

The forced swimming test (FST), which evaluates depression-like behaviors through the measurement of the duration of immobility, serves as a stress-induced avoidance reduction assessment. The duration of immobility was notably higher in the M-group in comparison to the C-group, suggesting an increased sense of desperation under stressful conditions (Figure 2F). Conversely, both the HCB-group LD and HCB-group HD demonstrated substantial decreases in immobility time when contrasted with the M-group (*p* < 0.01, *p* < 0.001).

The sucrose preference test (SPT) is commonly used to reflect anhedonia in mice, a key symptom of depression. The percentage of sucrose preference in the M-group was significantly lower than in the C-group (*p* < 0.001), indicating marked anhedonia. Both the HCB-group LD and HCB-group HD reversed this decrease in sucrose preference (*p* < 0.001) (Figure 2G). Fluoxetine (Flx), a commonly used antidepressant, also demonstrated significant effects in recovering activity and cognitive ability [14].

Subsequently, neurotransmitter and neurotrophic factor levels were measured across different groups using ELISA. As shown in Figure 2H,I, the levels of 5-HT and DA in the hippocampi were significantly lower in the M-group compared to the C-group, aligning with findings in clinical depression patients [15,16]. In the HCB treatment group, both serotonin (5-HT) and dopamine (DA) concentrations were elevated in the HD group compared to the M group (*p* < 0.001, *p*< 0.01), a statistically significant difference. These findings are consistent with the pharmacologic effects observed in the positive drug group (Flx group). The results of this study provide further evidence to support the potent antidepressant properties of HCB.

Brain-derived neurotrophic factor (BDNF), an important neurotrophic factor, plays a critical role in neuronal survival and function [17,18]. The expression level of BDNF was lower in the M-group compared to the C-group, while the HCB-group HD exhibited a higher BDNF level than the M-group (*p* < 0.01) (Figure 2J).

Pathological changes were further evaluated using Nissl staining (Figure 2L). Nissl bodies, which can degrade or disappear under prolonged stress or damage, are commonly used to assess neuronal injury [19]. As shown in Figure 2K, the number of Nissl bodies in the hippocampal CA3 region was significantly decreased in the M-group compared to the C-group (*p* < 0.001), indicating structural damage to neurons in the hippocampi of the M-group. However, the HCB-group HD significantly recovered the number of Nissl bodies in the CA3 region compared to the M-group (*p* < 0.001). The destruction of neurons caused by CUMS was notably alleviated in the HCB-group HD.

Overall, these results demonstrate that HCB exerts considerable therapeutic effects on CUMS-induced depression.

### 2.3. Network Pharmacology Analysis of HCB’s Interaction with Depression

**Figure 3:**
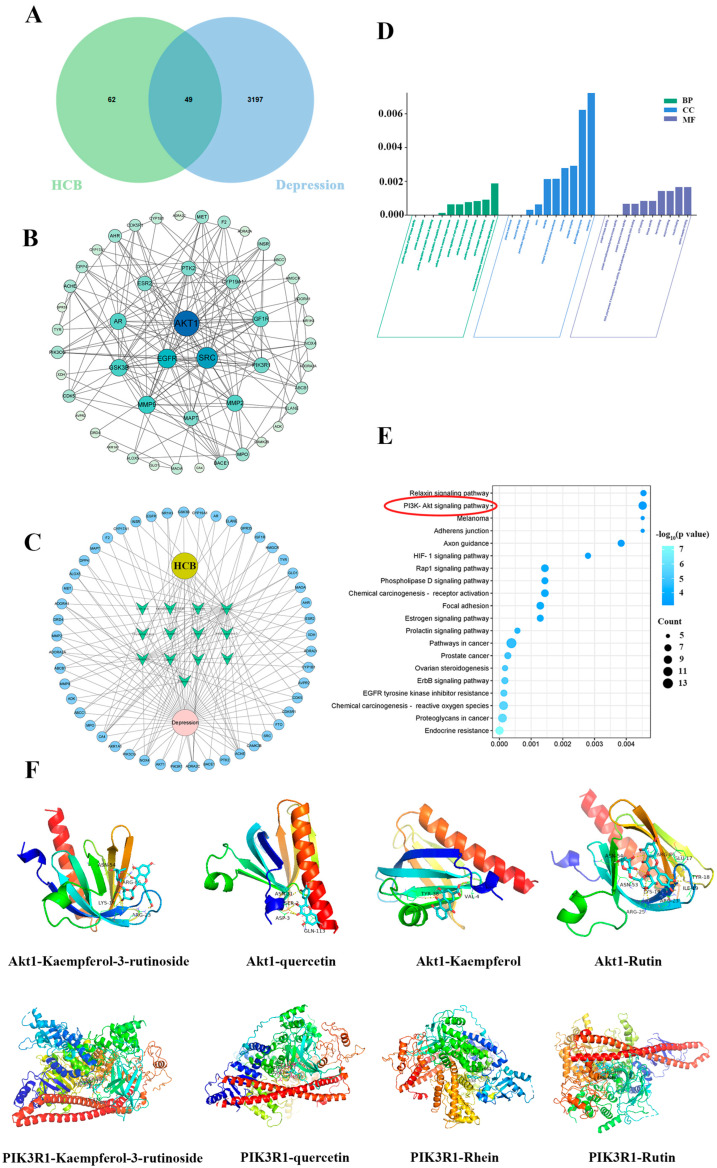
Analysis results of network pharmacology and molecular docking. (**A**) Venn mapping of HCB on depression. (**B**) PPI networks of candidate targets. (**C**) The network construction of compounds–targets–diseases. (**D**) GO enrichment analysis. (**E**) KEGG pathway analysis. (**F**) Molecular docking diagram of active constitutes and potential targets.

Based on the identification of 62 constituents in HCB, target predictions for these chemical constituents were obtained from the Swiss Target Prediction database, while 3197 targets related to depression were retrieved from the GenClip3 database. The combination of active constituents and disease targets revealed 49 overlapping targets (Figure 3A), leading to the identification of 13 potential active chemical constituents in HCB (Table 2). The protein–protein interaction (PPI) network of these potential targets, generated using STRING, is shown in Figure 3B. As illustrated in Figure 3C, the overlapping targets constructed a multilateral network, demonstrating that individual compounds can interact with multiple targets and that various compounds can act on a common target.

According to Table S1, the top 10 potential treatment targets of HCB were identified as Akt1, SRC, EGFR, MMP9, GSK3B, AR, MMP2, IGF1R, PIK3R1, and MAPT, which may be associated with HCB’s antidepressive mechanism through cellular functions, such as proliferation, survival, and apoptosis [20]. GO analysis revealed 211 biological processes (BP), 47 cellular components (CC), and 65 molecular functions (MF), from which the top 10 were selected for visual analysis. Additionally, 84 KEGG pathways were identified (Figure 3E), highlighting key signaling pathways, such as the Relaxin signaling pathway and the PI3K/Akt signaling pathway, which are linked to cell information processing, body systems, and human diseases. Notably, the PI3K/Akt signaling pathway, involved in processes like proliferation, survival, and apoptosis, suggests that HCB may regulate cellular functions in the treatment of depression.

To validate these findings, target proteins PIK3R1 and Akt1 were selected for molecular docking analysis (Figure 3F). The binding energies between the chemical constituents and targets, as indicated in Table S2, were below zero, suggesting that the molecular interactions are potentially spontaneous and stable.

### 2.4. Potential Mechanism of HCB on Depression by RNA Sequencing

**Figure 4:**
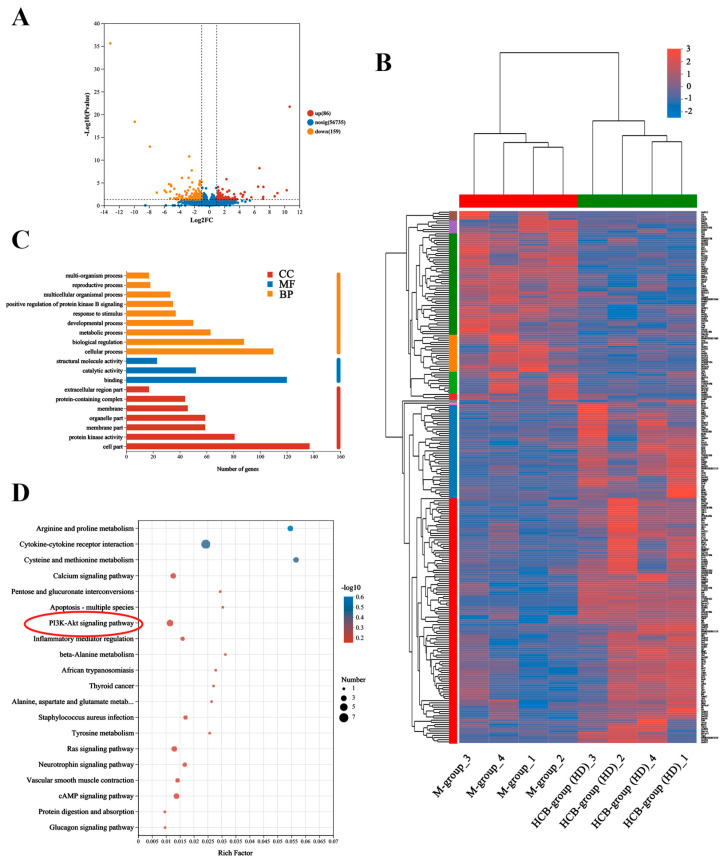
RNA sequencing analysis of hippocampus. (**A**) Volcano map of DEGs. (**B**) Hierarchical clustering analysis of DEGs. (**C**) Functional annotation analysis of GO using DEGs. (**D**) Functional enrichment analysis of KEGG using DEGs.

To investigate the molecular mechanisms underlying HCB intervention in a depression model induced by CUMS, an analysis of RNA sequencing on hippocampal tissues was performed. The number of differentially expressed genes (DEGs) was determined using DESeq2. A total of 245 DEGs were identified, including 86 upregulated and 159 downregulated genes between the M-group and the HCB-treated group (HD) (*p* < 0.05, fold change > 2) (Figure 4A). The observed pathological and molecular alterations within the hippocampus exhibited a notable alignment with the hierarchical clustering patterns of the DEGs. This consistency suggests a correlation between these changes and the specific gene expression profiles (Figure 4B).

Next, we examined the differential molecules and related pathways between the M-group and the HCB-treated group (HD). GO enrichment analysis revealed that DEGs were involved in nine biological processes (BP), three molecular functions (MF), and seven cellular components (CC), suggesting that the Akt signaling pathway, metabolic processes, developmental processes, and reproductive processes may play key roles in the treatment of depression (Figure 4C).

Furthermore, the analysis of KEGG pathway enrichment indicated that DEGs were mainly concentrated in pathways associated with the metabolism of arginine and proline, interactions between cytokines and their receptors, as well as the PI3K/Akt signaling pathway—pathways associated with abnormal cellular proliferation, survival, and apoptosis. Remarkably, these findings were consistent with the results of the network pharmacology analysis (Figure 4D).

### 2.5. HCB Regulates the PI3K/Akt/CREB Signaling Pathway

The results from both network pharmacology and RNA sequencing suggest that the molecular mechanism by which HCB regulates CUMS-induced depression is closely related to cellular proliferation, survival, and apoptosis. Additionally, the PI3K/Akt signaling pathway, which is strongly associated with these cellular processes, was identified through KEGG pathway enrichment analysis from both network pharmacology and RNA sequencing data.

**Figure 5:**
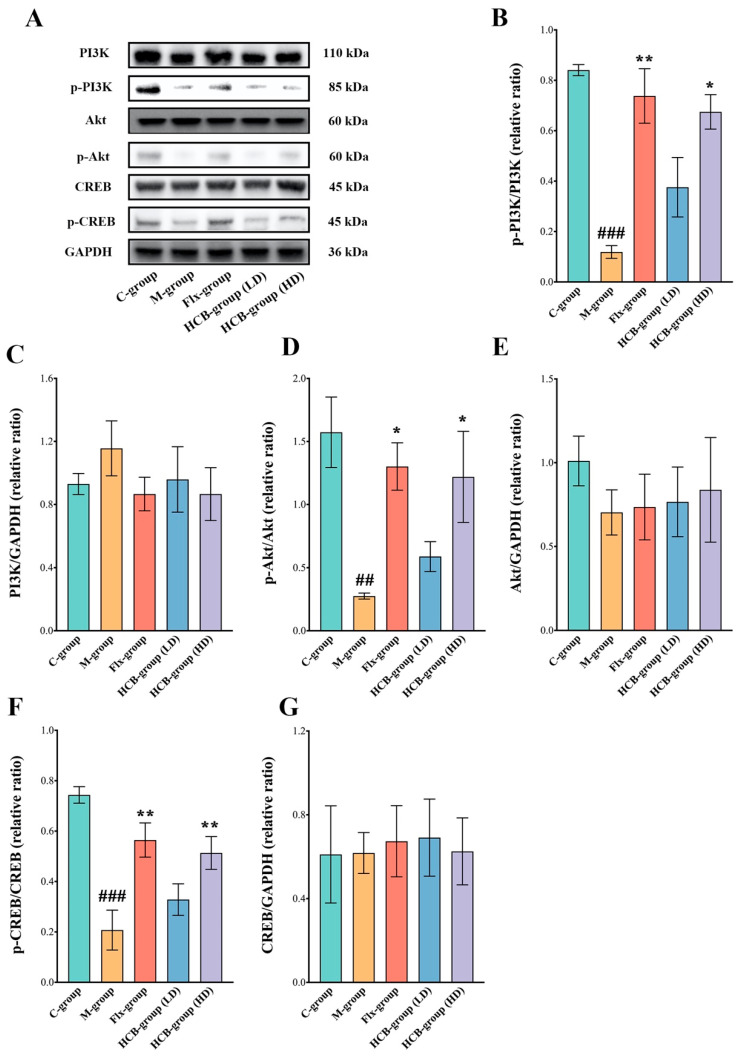
HCB regulated PI3K/Akt/CREB signaling pathway (**A**) Representative protein bands of PI3K, p-PI3K, Akt, p-Akt, CREB, and p-CREB in hippocampal. Statistical graphs of relative protein expression of ratio of p-PI3K/PI3K (**B**), PI3K/GAPDH (**C**), p-Akt/Akt (**D**), Akt/GAPDH (**E**), p-CREB/CREB (**F**), and CREB/GAPDH (**G**). Data are presented as mean ± SEM, ^##^ *p* < 0.01, ^###^ *p* < 0.001 vs. control group (C-group); * *p* < 0.05, ** *p* < 0.01 vs. model group (M-group).

As shown in Figure 5A, the involvement of the PI3K/Akt signaling pathway was further confirmed by Western blot analysis. The expression levels of phosphorylated PI3K (p-PI3K) relative to total PI3K and phosphorylated Akt (p-Akt) relative to total Akt in the hippocampi of the M-group were significantly lower than in the C-group (*p* < 0.001). The expression level of p-PI3K/PI3K in the HCB-treated group (HD) was significantly increased compared to the M-group (*p* < 0.05) (Figure 5B,C). Similarly, the ratio of p-Akt/Akt in the HCB-treated group (LD, HD) was markedly higher than that in the M-group (*p* < 0.05) (Figure 5D,E).

To further investigate the potential mechanism of HCB’s antidepressant effect, we examined the expression level of the important downstream protein CREB in the hippocampi of mice. CREB expression in the M-group was significantly lower than in the C-group (*p* < 0.001). However, treatment with HCB (LD, HD) significantly increased CREB expression compared to the M-group (*p* < 0.01) (Figure 5F,G).

These findings indicate that HCB may alleviate CUMS-induced depression by regulating the PI3K/Akt/CREB signaling pathway, thereby promoting protein synthesis and influencing neuronal proliferation and growth.

## 3. Discussion

Depression, a condition characterized by high morbidity and a profound impact on mental health, affects millions of individuals worldwide. This mental health disorder not only poses a significant threat to public health but also increases the potential risk of medical malpractice if inadequately addressed. With the rising global burden of depression, especially in regions with constrained healthcare resources, improving treatment approaches is a pressing priority. Effective therapeutic options that are both accessible and affordable are urgently needed to alleviate the heavy social and economic toll of depression. Currently, there are two primary applications of HCB, also known as daylily. Fresh HCB is widely recognized as a popular ornamental flower but is inedible due to the presence of toxic components [21]. In contrast, dried HCB is both a widely consumed vegetable and a traditional Chinese medicinal herb [22]. Recent studies have highlighted the therapeutic potential of HCB in treating depression, but the connection between the active constituents of HCB and its underlying mechanisms in the treatment of depression has been largely unexplored.

The hippocampus, a key brain region associated with emotion regulation, plays a central role in both depression and anxiety [23]. Chronic stress has been shown to inhibit hippocampal neurogenesis and promote apoptosis in hippocampal cells [24]. Clinical studies have reported hippocampal atrophy in individuals diagnosed with depression [25], further highlighting the critical role of this brain region in the pathophysiology of the disorder. The PI3K/Akt/CREB signaling pathway, extensively studied in the context of neuronal survival and neuroplasticity, has been found to be closely linked to the development of depression [26]. Previous research has demonstrated that the activity of PI3K, and Akt is significantly reduced in individuals with depression [27]. Moreover, in CUMS-induced mice, the ratios of phosphorylated PI3K (p-PI3K) to total PI3K and phosphorylated Akt (p-Akt) to total Akt are decreased, indicating that the PI3K/Akt signaling pathway plays a crucial role in regulating depression induced by chronic stress [28].

CREB (cAMP response element-binding protein) is a vital downstream effector of the PI3K/Akt pathway and serves as a key transcription factor involved in neurogenesis and synaptic plasticity. CREB regulates the expression of brain-derived neurotrophic factor (BDNF), a critical neurotrophin that supports neuronal growth, survival, and differentiation, particularly in the hippocampus [29,30]. BDNF plays a pivotal role in maintaining synaptic plasticity and promoting neuronal regeneration. A deficiency of BDNF has been linked to disruptions in the synthesis of neurotransmitters, such as serotonin (5-HT) and dopamine (DA), which are essential for emotional regulation. Reduced levels of BDNF are thought to contribute to the onset and progression of depression [31]. Therefore, the PI3K/Akt/CREB signaling pathway is of paramount importance in mitigating depression by promoting neurogenesis and enhancing neuronal survival [32].

**Figure 6:**
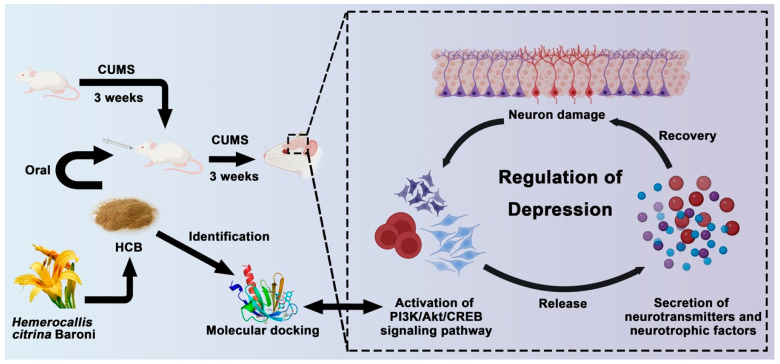
Identification of antidepressant constitutes in HCB and its underlying mechanism on the treatment of depression.

In this study, we characterized the chemical composition of ethanol-extracted HCB using ultra-high performance liquid chromatography-Q-orbitrap HRMS, and we identified a total of 13 active constituents in HCB that contribute to its antidepressant effects, including flavonoids, such as rutin, chrysin, kaempferol, and quercetin. Flavonoids have well-known neuroprotective properties that are similar to those of neurotrophic factors (NTFs), such as BDNF. Several studies have shown that flavonoids can improve neuronal survival and function by modulating BDNF production, particularly through the activation of the PI3K/Akt signaling pathway [33]. These compounds also promote synaptic plasticity, neuronal growth, and differentiation, making them promising candidates for the treatment of depression. Their antidepressant pharmacological effects were subsequently demonstrated using the chronic unpredictable mild stress (CUMS) depression model. The results of this study provide strong evidence for the potential of HCB as a therapeutic agent for depression through behavioral experiments on CUMS mice, histological analysis using Nissl staining, and the detection of relevant biochemical indices by ELISA. These experimental results demonstrate the practical significance of HCB in the treatment of depression. In addition, through network pharmacology, we predicted the potential molecular targets of HCB for the treatment of depression and identified the PI3K/Akt signaling pathway as a key target. Molecular docking analysis confirmed that the major chemical components of HCB have strong binding affinities with their respective target proteins, further supporting our hypothesis that HCB exerts its antidepressant effects by regulating cell proliferation and apoptosis through the PI3K/Akt signaling pathway. In addition to virtual validation, we also performed RNA sequencing of hippocampal tissues from CUMS mice to further investigate the molecular mechanisms involved. Our sequencing results confirmed that the PI3K/Akt signaling pathway plays a crucial role in the development of depression, and there were significant differences in the expression of key target genes between the normal and CUMS model groups. In addition, the expression of these target genes changed significantly after treatment with HCB, which is consistent with the results derived from network pharmacology. The convergence of data from network pharmacology, molecular docking, and RNA sequencing strengthens the validity of our findings and provides a comprehensive understanding of how HCB exerts its role in the treatment of depression. The combined application of network pharmacology and transcriptomics has proven to be an efficient and powerful approach to study the potential mechanisms of multicomponent natural products in the prevention and treatment of various diseases. Finally, during the wet experiments, we confirmed that HCB has the ability to regulate the PI3K/Akt/CREB signaling pathway, which has rarely been reported in previous studies. Our results showed that HCB increased the ratios of p-PI3K/PI3K, p-Akt/Akt, and p-CREB/CREB in the hippocampus of CUMS-induced mice. These findings suggest that the antidepressant effect of HCB may be mediated through the PI3K/Akt/CREB pathway, which is a key molecular cascade for neuronal growth, survival, and apoptosis, plays an important role in the promotion of hippocampal neurogenesis and neuronal survival, and is most likely one of the main mechanisms for the antidepressant effect of HCB, as previously described. It is highly likely that it is one of the main mechanisms by which HCB exerts antidepressant effects. In summary, 62 chemical components were identified from HCB using ultra-high performance liquid chromatography-Q-orbitrap HRMS, of which 13 were identified as potentially active components targeting 49 depression-related proteins. In addition, it was found that HCB significantly attenuated cognitive impairment, anxiety-like behavior, and dysphoria-like behavior. In the hippocampal CA3 region, the expression levels of 5-hydroxytryptamine (5-HT), dopamine (DA), and brain-derived neurotrophic factor (BDNF) were elevated. Network pharmacology and transcriptomic findings suggest that the PI3K/Akt/CREB signaling pathway is critical for the therapeutic efficacy of HCB in depression. Molecular biology studies confirmed that HCB increased the expression levels of p-PI3K/PI3K, p-Akt/Akt, and p-CREB/CREB (Figure 6).

However, it has to be recognized that the study in this paper has some limitations. The first is the limitation of the depression animal model itself, which is mainly reflected in the following aspects. The first aspect is that there are too many complex factors in the modeling process, and it is impossible to verify whether the model is finally formed or not. The second aspect is that the behavior of experimental animals is unpredictable and prone to variability, so the behavioral analysis based on the behavior of experimental animals has a large error. Finally, the operation procedures of CUMS have not been standardized, because CUMS involves multiple stresses and emphasizes “randomness”, so the operation procedures of CUMS in different laboratories are almost different, and how to establish standard operation procedures has become a key issue in the development of the model. The second is the limitation of experimental results based on computational predictions. In reality, intermolecular interactions are very complex, including electrostatic interactions, van der Waals forces, hydrogen bonding, and many other forces. However, the force field parameters are often average values or idealized models obtained based on experimental data and theoretical calculations, which cannot accurately describe the real forces on each atom in various complex environments. Meanwhile, the protein flexibility is not sufficiently considered in the simulation process, which affects the accurate prediction of the binding mode and binding affinity. In addition, at present, this paper only validates the effectiveness of the unit drug, and further validation of single compounds is needed in the follow-up, which would help to expand the mechanism and clarify the specific regulatory mechanism and binding site of HCB on the PI3K/Akt/CREB signaling pathway, etc. Meanwhile, the histology technology should be updated, and spatial RNA sequencing (spRNA-seq) is an emerging and transformative technology. spRNA-seq is able to provide RNA-seq data with spatial locations. Understanding this complex spatial information greatly helps us to clarify how molecules and sites communicate with each other. spRNA-seq is still in its infancy, and it is believed that, as the technology continues to advance, more definitive proofs of the relevant mechanisms can be achieved.

## 4. Materials and Methods

### 4.1. Materials and Chemicals

HCB was purchased from Feng County, Shanxi Province, China. Acetonitrile (LC/MS reagent grade) was obtained from Thermo Fisher Scientific Inc. (Beijing, China). Experimental water was Wahaha pure water from Wahaha Co., Ltd. (Beijing, China). Formic acid, used as a modifier in the mobile phase, was procured from Tanmo Quality Inspection Technology Co., Ltd. (Beijing, China). Protein phosphatase inhibitor mixture, RIPA lysate, BCA kit, TBST, ECL hypersensitive luminescent liquid, dried skimmed milk, and 30% acrylamide were obtained from Beyotime Biotechnology Inc. (Shanghai, China). ELISA kits for 5-HT (ml001891-2), DA (ml002024-2), and BDNF (ml002219-2) were purchased from Enzyme-linked Biotechnology Co., Ltd. (Shanghai, China). GAPDH (60004-1-IG), PI3K (27921-1-AP), and Akt (10176-2-AP) antibodies were purchased from Proteintech Group (Wuhan, China), while p-PI3K (17366s), p-Akt (4060T), CREB (9197T), p-CREB (9198s), and antirabbit IgG HRP-conjugated antibodies (7074s) were obtained from Cell Signaling Technology (Beverly, MA, USA).

### 4.2. Preparation of HCB

HCB (10 g) was placed in a nonwoven bag, and 100 mL of 70% ethanol was added to a 500 mL round-bottom flask. The flask was heated for 1.5 h in a water bath at 80 °C. After removing the ethanol by rotary evaporation, the aqueous solution was obtained and, subsequently, freeze-dried to yield ethanol extract HCB powder. The ethanol extract powder (10 mg) was weighed accurately and dispersed in 1 mL of 70% methanol. This was sonicated for 10 min using an Elmasonic P 300H ultrasonic cleaning unit (Elma, Singen, Germany). The sample was filtered using a 0.2 μm syringe filter (Port Washington, NY, USA) for UHPLC-Q-Orbitrap HRMS. All sample preparations were carried out at 4 °C in the dark.

### 4.3. UHPLC-Q-Orbitrap HRMS Analysis

The chemical constituents of HCB were analyzed using UHPLC-Q-Orbitrap HRMS, following the method described in a previous study [34]. Chemical constituents in HCB were investigated by using the UHPLC-Q-Orbitrap-HRMS. The mobile phase was composed of water/0.1% formic acid (A) and acetonitrile (B). The elution conditions were set as follows: 0–10 min, 4.0–20% B; 10–15 min, 20–40% B; 15–17 min, 40–70% B; 17–20 min, 70–90% B; 20–25 min, 90% B; 25–26 min, 90–4.0% B; and 26–30 min, 4.0% B. Full-scan data within the range of *m*/*z* (mass-to-charge ratio) 100–1500 were acquired.

### 4.4. Network Pharmacology Analysis

The active constituents of HCB and the potential targets for treating depression were investigated using network pharmacology [35]. The compounds in HCB were identified. Before forecasting, the “Canonical SMILES” of each compound were acquired from PubChem (http://bioinfo.org/kobas/ accessed on 13 September 2023). Targets of compounds in HCB were predicated with their “Canonical SMILES” on the Swiss Target Prediction database (http://swisstargetprediction.ch/ accessed on 13 September 2023). To analyze the therapeutic targets of depression in scientific texts deposited in MEDLINE database, a data-mining analysis of scientific literature was performed using the GenClip3 (http://cismu.net/genclip3/analysis.php, accessed on 13 September 2023) web service. The venn diagrams were produced by using the online software Venny.2.1 to show the number of targets linked to depression and identified compounds. Then, the intersection was exported to R software 4.4.2. Then, the software Cytoscape 3.9.1 was used to construct the “compounds–targets–diseases” network. Protein–protein interactions (PPI) in human genome were extracted from version 11.5 of STRING (https://STRING-db.org/, accessed on 13 September 2023), a weighted interaction database containing physical and functional interactions that are integrated from multiple data sources. Based on the above analyses, the intersecting targets of HCB and antidepression were used to build the PPI network. Subsequently, the overlap targets were submitted to STRING tool to acquire PPI relationships with the species limited to “Homo sapiens”. In order to construct a PPI network with high confidence edges, we filtered the STRING with the threshold 0.7. Only interactions with a weight above the threshold were selected for the newly constructed PPI network. Finally, Cytoscape 3.9.1 was used to visualize the PPI network. The three topological properties, “degree”, “betweenness”, and “closeness”, were calculated to screen the putative targets for topological importance. Gene Ontology (GO) and Kyoto Encyclopedia of Genes and Genomes (KEGG) pathway enrichment analysis were used. To clarify the pathways that are involved in putative HCB targets, DAVID 6.8 (https://david.ncifcrf.gov, accessed on 19 September 2023) was used to perform the GO and KEGG enrichment. In the study, first step, a gene list was entered into the search box; subsequently, the identifier “OFFICIAL GENE SYMBOL” was selected, the list type “Gene List” was chosen, and then the list was submitted. The second step, “Homo sapiens” was selected to limit annotations and “List 1” selected. In the third step, the background of “Homo sapiens” was selected. In the last step, the “Functional Annotation Chart” was selected to obtain GO (“GOTERM-BP-DIRECT”) and KEGG (“KEGG-PATHWAY”) pathway analysis results. Finally, the functional categories were identified and ranked by *p*-values, and those GO terms and KEGG pathways with *p*-value ≤ 0.001 were recognized as significant.

### 4.5. Molecular Docking

Potential targets were selected for molecular docking with the screened constituents. The 3D structures of the constituents were downloaded from PubMed, while those of the potential targets were obtained from the RCSB protein structure database [36]. Molecular docking was performed using AutoDock 1.5.7 software.

### 4.6. Animals

ICR male mice (18–22 g, 7 weeks old) were obtained from Beijing Vital River Bioscience Limited Company’s Laboratory Animal Center (Beijng, China). All animal-related experimental procedures were carried out in accordance with the Guide for the Institutional Animal Care and Use Committee (IACUC) and approved by Beijing University of Chinese Medicine Animal Care Committee (Registration number: BUCM-2022032402-1166). The animals were allowed to acclimate to the laboratory environment for one week. All care and experimental procedures were approved by the Ethics Committee of Beijing University of Chinese Medicine.

### 4.7. CUMS Model and Grouping

The CUMS model was established following a previous report [37]. Weekly stressors included 24 h food or water deprivation, 5 min swims in ice water (4 °C), 4 h restraint, 10 min walks on ice (4 °C), 6 h exposure to noise and flashing lights, and 12 h exposure to all-night lights (Table S3). These stressors were randomly assigned throughout the week and repeated for six weeks. Mice were divided into five groups (*n* = 8 per group): control (C-group), model (M-group), fluoxetine (Flx-group, 3 mg/kg b.w.), HCB low-dose (HCB-group LD, 112.5 mg/kg b.w.), and HCB high-dose (HCB-group HD, 450 mg/kg b.w.). Dosages and administration times for HCB were based on previous studies [38]. The C-group received no treatment, with a normal diet and water. After three weeks of modeling, drug intervention began, with daily administration for three weeks. HCB extract powders were dissolved in distilled water for intragastric administration, with the dosage and volume adjusted weekly, according to weight changes.

### 4.8. Behavioral Tests

Depressive behaviors in mice were assessed using the open field test (OFT), forced swimming test (FST), and sucrose preference test (SPT), slightly modified from previously reported methods [39]. The mice were deprived of food and water 24 h before SPT. Two bottles filled with distilled water and 1% sucrose solution, which were weighted and marked in advance, were given to the mice for 1 h. The consumption of sucrose solution and distilled water was recorded after 1 h. Then, the two bottles were removed simultaneously and measured. The sucrose preference was calculated by the amount of sugar water consumed/the total amount consumed. OFT provided simultaneous measures of movement, exploration, and anxiety. An open box (50 cm × 50 cm × 50 cm) was placed in a quiet room, the black floor was divided into 16 equal-sized squares and four side walls, and a computer above the middle of the box was connected to a video camera. The mice were placed in the center of the experimental device and explored freely for 5 min. After each experiment, the feces were cleaned with 75% ethanol and the experimental apparatus was wiped. The distance moved, cumulative duration in the center, and not moving time were tracked and measured by the software EthoVision XT 9. FST was performed in a cylinder with a height of 30 cm × a diameter of 17 cm, and the mice were placed in it. The cylinder contained 25 ± 2 °C of water with a depth of about 15 cm, so that the mice could not support their bodies with their feet touching the bottom. FST was performed for 4 min after 2 min adaption, and the mice were trained to learn to swim before FST 24 h. The immobility time during the 4 min was recorded.

### 4.9. Preparation of Tissue Samples

Following behavioral tests, mice were sacrificed by decapitation. Eyeball blood was coagulated for 10 min at 4 °C, then centrifuged to separate the serum, which was stored at −20 °C. The whole brain was fixed in 4% paraformaldehyde (PFA) for Nissl staining, while the hippocampi were collected and stored in frozen tubes at −80 °C for ELISA and Western blot analysis.

### 4.10. Nissl Staining

The Nissl staining of the hippocampus tissues was performed using standard methods [40]. For Nissl staining, brain sections were dewaxed with xylene, hydrated with 75–90% graded alcohol, stained with Nissl solution for 10 min, washed with distilled water, dehydrated with anhydrous ethanol, transparentized with xylene, and fixed with neutral balm. Whole tissues in images were observed and photographed with a digital slide scanner under 10× microscopy to identify Nissl bodies, select areas to be observed, and take 40× pictures to count Nissl bodies.

### 4.11. ELISA Test

Levels of 5-HT, DA, and BDNF in hippocampal tissues were measured by ELISA, following the manufacturer’s instructions. A total of 50 μL of standards and samples were added into the appropriate wells, and nothing was added to the blank well. Then, 100 μL of enzyme conjugate was added to standard wells and sample wells except the blank well, covered with an adhesive strip, and incubated for 60 min at 37 °C. After washing the microtiter plate 4 times, substrate A and B were added into each well and incubated for 15 min at 37 °C with protection from light. Finally, the optical density (O.D.) was read at 450 nm using a microtiter plate reader within 15 min of adding 50 μL stop solution into each well.

### 4.12. Western Blot

Western blot analysis was conducted to assess the protein levels of p-PI3K, PI3K, p-Akt, Akt, p-CREB, and CREB. The expression levels of p-PI3K, PI3K, p-Akt, Akt, p-CREB, and CREB were measured by Western blot analysis. The total protein of the hippocampal tissues was extracted using RIPA Lysis buffer. After the loading buffer was mixed and heated at 100 °C for 5 min, the proteins were separated on the 4–12% SDS-PAGE gels at 30 μg and transformed onto a PVDF membrane. The PVDF membranes were blocked using the 5% non-fat dry milk in 0.05% TBST and then were incubated at 4 °C overnight with primary antibodies. The PVDF membranes were washed with TBST for 5 min three times, followed by incubation with secondary antibody conjugated to horseradish peroxidase for 1 h at room temperature. The PVDF membranes were washed in the same way, and protein bands were detected by ECL hypersensitive luminescent liquid.

### 4.13. RNA Sequencing of the Hippocampus

Total RNA was extracted from the tissue using TRIzol^®^ Reagent, according to the manufacturer’s instructions (Invitrogen, Carlsbad, CA, USA), and genomic DNA was removed using DNase I (TaKara, Kusatsu, Japan). RNA quality was determined by 2100 Bioanalyser (Agilent, Santa Clara, CA, USA) and quantified using the ND-2000 (NanoDrop Technologies, Wilmington, DE, USA). Only high-quality RNA samples (OD260/280 = 1.8~2.2, OD260/230 ≥ 2.0, RIN ≥ 6.5, 28S:18S ≥ 1.0, >1 μg) were used to construct sequencing library.

A RNA-seq transcriptome library was prepared following the TruSeqTM RNA sample preparation Kit from Illumina (San Diego, CA, USA) using 1 μg of total RNA. Shortly, messenger RNA was isolated according to the polyA selection method by oligo (dT) beads and then fragmented by fragmentation buffer. Secondly, double-stranded cDNA was synthesized using a SuperScript double-stranded cDNA synthesis kit (Invitrogen, CA) with random hexamer primers (Illumina). Then, the synthesized cDNA was subjected to end-repair, phosphorylation, and ‘A’ base addition, according to Illumina’s library construction protocol. Libraries were size-selected for cDNA target fragments of 300 bp on 2% Low Range Ultra Agarose followed by PCR amplified using Phusion DNA polymerase (NEB) for 15 PCR cycles. After being quantified by TBS380, the paired-end RNA-seq sequencing library was sequenced with the Illumina HiSeq xten/NovaSeq 6000 sequencer (2 × 150 bp read length).

The raw paired-end reads were trimmed and quality-controlled by SeqPrep (https://github.com/jstjohn/SeqPrep accessed on 13 September 2023) and Sickle (https://github.com/najoshi/sickle accessed on 13 September 2023) with default parameters. Then, the clean reads were separately aligned to reference genome with orientation mode using HISAT2 (http://ccb.jhu.edu/software/hisat2/index.shtml accessed on 13 September 2023) software. The mapped reads of each sample were assembled by StringTie (https://ccb.jhu.edu/software/stringtie/index.shtml?t=example accessed on 13 September 2023) in a reference-based approach. Then, R studio was used for downstream bioinformatics analysis. Differential expression gene was extracted with the Limma package [41].

To identify DEGs (differential expression genes) between two different samples, the expression level of each transcript was calculated according to the transcripts per million reads (TPM) method. RSEM (http://deweylab.biostat.wisc.edu/rsem/ accessed on 13 September 2023) was used to quantify gene abundances. Essentially, differential expression analysis was performed using the DESeq2/DEGseq/EdgeR with Q value ≤ 0.05, and DEGs with |log2FC| > 1 and Q value ≤ 0.05(DESeq2 or EdgeR)/Q value ≤ 0.001(DEGseq) were considered to be significantly different expressed genes. In addition, functional enrichment analysis, including GO and KEGG, was performed to identify which DEGs were significantly enriched in GO terms and metabolic pathways at a Bonferroni-corrected *p*-value ≤ 0.05 compared to the whole-transcriptome background. GO functional enrichment and KEGG pathway analysis were carried out by Goatools (https://github.com/tanghaibao/Goatools accessed on 13 September 2023) and KOBAS (http://bioinfo.org/kobas/ accessed on 13 September 2023).

### 4.14. Statistical Analysis

The data collected in this study were presented as mean values along with their standard error of the mean (SEM) to provide a clear understanding of the central tendency and variability within the dataset. Statistical analyses were conducted using SPSS software (version 20.0). To assess statistical significance, Student’s *t*-test was employed for comparing two independent samples, allowing for a straightforward evaluation of differences between these groups. In instances where comparisons involved three or more groups, a one-way analysis of variance (ANOVA) was utilized, enabling a more comprehensive assessment of the data across multiple categories. For the purposes of this study, a threshold for statistical significance was determined at *p* < 0.05, indicating a likelihood of less than 5% that the observed differences occurred by chance.

## 5. Conclusions

This study identified 62 chemical constituents in HCB and demonstrated its potential to alleviate depression through the modulation of the PI3K/Akt/CREB signaling pathway. These findings suggest that HCB could serve as a valuable candidate for the development of therapeutic agents, reinforcing its dual role as a medicinal and functional food source in the context of antidepressant interventions.

## Figures and Tables

**Table 1:** Information on the constituents identified in dried flowers of HCB in positive ionization mode.

Constituents	Formula	Retention Time (min)	Identity	Precursor Ion			Fragment Ions (*m*/*z*)
				Experimental (*m*/*z*)	Theoretical (*m*/*z*)	Mass Accuracy (△ppm)	
Clionasterol	C_29_H_50_O	0.32	[M+H]^+^	415.3934	415.3933	−0.24	397.3834, 179.1434, 123.1168
Quinic acid	C_7_H_12_O_6_	1.43	[M+H]^+^	193.0708	193.0707	0.52	175.0600, 157.0494, 133.0495, 115.0389
Kwansonine A	C_16_H_26_N_2_O_11_	1.44	[M+H]^+^	423.1609	423.1609	0.00	261.1081, 245.1131, 243.0973, 217.1178, 202.1068, 163.0712, 146.0447, 145.0607, 144.0658
Oxypinnatanine	C_10_H_16_N_2_O_6_	1.50	[M+H]^+^	261.1083	261.1081	0.77	246.0971, 245.1132, 230.1023, 215.1021, 200.0911, 187.1077, 163.0712, 146.0447, 130.0498
Syringic acid	C_9_H_10_O_5_	1.97	[M+H]^+^	199.0602	199.0601	0.50	183.0652, 169.0494, 153.0541, 151.0389, 139.0388, 123.0439
Pinnatannine	C_10_H_16_N_2_O_5_	2.07	[M+H]^+^	245.1134	245.1132	0.82	228.0856, 227.1021, 128.0704, 100.0759, 84.0449
Oxypinnatanine A	C_10_H_16_N_2_O_5_	2.36	[M+H]^+^	245.1132	245.1132	0.00	230.1023, 228.0863, 215.1029, 201.1233, 198.0763, 186.1128, 156.1022, 144.0655, 116.0707
Longitubanine A	C_10_H_16_N_2_O_2_	2.40	[M+H]^+^	245.1130	245.1132	−0.82	227.1026, 210.0765, 201.1238, 172.0966, 163.0716, 156.1020, 145.0607, 120.0655, 100.0757
Adenosine	C_10_H_13_N_5_O_4_	2.67	[M+H]^+^	268.1040	268.1040	0.00	136.0620
Kwansonine B	C_16_H_26_N_2_O_10_	3.25	[M+H]^+^	407.1659	407.1660	−0.25	391.1699, 325.1236, 245.1129, 229.1178, 163.0711, 147.0762
Longitubanine B	C_10_H_16_N_2_O_4_	3.28	[M+H]^+^	229.1182	229.1183	−0.44	214.1070, 170.1172, 147.0764, 132.0655, 130.0499, 128.0704, 117.0545, 104.0704
Kwansonine C	C_16_H_26_N_2_O_10_	3.48	[M+H]^+^	407.1660	407.1660	0.00	391.1700, 307.1130, 245.1131, 229.1180, 163.0712, 145.0608
Fuluanine A	C_9_H_13_NO_5_	5.46	[M+H]^+^	216.0869	216.0866	1.39	198.0763, 186.0760, 118.0499
Vanillic acid	C_8_H_8_O_4_	5.49	[M+H]^+^	169.0497	169.0495	1.18	153.0547, 139.0390, 125.0598, 123.0440, 109.0648
Chlorogenic acid	C_16_H_18_O_9_	6.39	[M+H]^+^	355.1024	355.1024	0.00	337.0901, 165.0542, 163.0390, 145.0283, 137.0595, 135.0442, 117.0336
Cryptochlorogenic acid	C_16_H_18_O_9_	6.39	[M+H]^+^	355.1024	355.1024	0.00	337.0901, 165.0542, 163.0390, 145.0283, 137.0595, 135.0442, 117.0336
Neochlorogenic acid	C_16_H_18_O_9_	6.39	[M+H]^+^	355.1024	355.1024	0.00	337.0901, 165.0542, 163.0390, 145.0283, 137.0595, 135.0442, 117.0336
Salidroside	C_14_H_20_O_7_	6.88	[M+H]^+^	301.1280	301.1282	−0.66	285.1331, 153.0910, 149.0962, 139.0754, 123.0805, 107.0856
Icariside D2	C_14_H_20_O_7_	6.89	[M+H]^+^	301.1282	301.1281	0.00	283.1176, 265.1069, 235.0963, 139.0755, 107.0857
7-hydroxycoumarin	C_9_H_6_O_3_	6.93	[M+H]^+^	163.0386	163.0390	−2.45	147.0436, 145.0284, 135.0445,
1′,2′,3′,4′-tetraphydro-5′-deoxypinnatanine	C_10_H_20_N_2_O_4_	7.42	[M+H]^+^	233.1503	233.1496	3.00	216.1234, 215.1384, 146.0450, 84.0442, 73.0283
3-O-*p*-coumaroylquinic acid	C_16_H_18_O_8_	7.93	[M+H]^+^	339.1075	339.1064	0.29	323.1125, 247.0817, 193.0711, 175.0604, 165.0546, 157.0496, 143.0708, 139.0390, 121.0650
4-O-*p*-coumaroylquinic acid	C_16_H_18_O_8_	7.93	[M+H]^+^	339.1075	339.1064	0.29	247.0806, 193.0709, 175.0601, 165.0545, 157.0495, 147.0443, 139.0389, 121.0648, 101.0598
Quercetin-3,7-2-O-glucose	C_27_H_30_O_17_	8.20	[M+H]^+^	627.1568	627.1556	1.91	465.1039, 303.0493
Isoquercetin	C_21_H_20_O_12_	8.34	[M+H]^+^	465.1031	465.1028	0.65	303.0507, 127.0398
Quercetin 3-O-rutinoside-7-glucoside	C_33_H_40_O_21_	8.59	[M+H]^+^	773.2130	773.2135	0.65	627.1515, 611.1633, 465.1054, 303.0501
4-O-caffeoyl-quinic acid	C_16_H_18_O_9_	8.61	[M+H]^+^	355.1023	355.1024	−0.28	339.1080, 337.0914, 293.1022, 193.0712, 181.0497, 175.0603, 163.0390, 145.0287, 113.0596
Methyl chlorogenate	C_17_H_20_O_9_	8.75	[M+H]^+^	369.1180	369.1180	0.00	355.1023, 339.1080, 195.0653, 175.0604, 177.0545, 163.0385, 157.0497, 131.0706, 121.0650
3-O-feruloylquinic acid	C_17_H_20_O_9_	8.75	[M+H]^+^	433.1133	433.1129	0.92	271.0601, 153.0181, 127.0389
Gallic acid	C_7_H_6_O_5_	9.21	[M+H]^+^	171.0286	171.0288	−1.17	153.0179, 137.0237, 127.0389, 109.0281
Hemerocallone	C_18_H_14_O_6_	9.87	[M+H]^+^	327.0871	327.0863	2.44	165.0549, 163.0752, 137.0601, 127.0391
Puerarin	C_21_H_20_O_9_	10.08	[M+H]^+^	417.1176	417.1180	−0.96	255.0647, 165.0544, 163.0392, 149.0599, 139.0388, 123.0442
2-hydroxychrysophanol	C_15_H_10_O_5_	10.24	[M+H]^+^	271.0601	271.0601	0.00	243.0648, 215.0704, 153.0548, 135.0442, 125.0599, 109.0648
Aloe emodin	C_15_H_10_O_5_	10.32	[M+H]^+^	271.0602	271.0601	0.37	243.0658, 137.0596, 123.0441, 107.0490
Kwanzoquinone G	C_16_H_10_O_6_	10.34	[M+H]^+^	299.0550	299.0550	0.00	271.0601, 243.0648, 165.0548, 147.0441, 137.0596
4-O-caffeoylshikimic acid	C_16_H_16_O_8_	10.60	[M+H]^+^	337.0912	337.0918	−1.78	319.0812, 229.0708, 185.0812, 181.0496, 174.0530, 159.0657, 149.0956, 131.0705, 111.0440
Ferulic acid	C_10_H_10_O_4_	10.62	[M+H]^+^	195.0661	195.0652	4.69	177.0554, 149.0590, 125.0598, 95.0496, 79.0540
Phenethyl-*β*-D-glu	C_14_H_20_O_6_	11.48	[M+H]^+^	285.1329	285.1333	−1.40	249.1117, 181.1222, 149.0962, 147.0806, 123.0805, 105.0699
Catechin	C_15_H_14_O_6_	12.35	[M+H]^+^	291.0858	291.0863	–1.72	183.0653, 169.0860, 167.0704, 153.0909, 137.0962, 109.0649
Chrysoobtusin	C_17_H_14_O_5_	12.61	[M+H]^+^	299.0913	299.0914	−0.33	271.0967, 181.0859, 151.0756, 123.0440, 121.0649
Quercetin	C_15_H_10_O_7_	12.68	[M+H]^+^	303.0499	303.0499	0.00	287.0547, 179.0336, 155.0338, 139.0388, 123.0439, 105.0699
Rutin	C_27_H_30_O_16_	12.71	[M+H]^+^	611.1627	611.1607	3.27	465.1049, 449.1090, 303.0499
Rhein	C_15_H_8_O_6_	12.72	[M+H]^+^	285.0394	285.0394	0.00	257.0438, 229.0501, 167.0337, 151.0387, 139.0390, 123.0441
Hyperoside	C_21_H_20_O_12_	13.21	[M+H]^+^	465.1021	465.1028	−1.51	303.0496
Kaempferol	C_15_H_10_O_6_	13.66	[M+H]^+^	287.0541	287.0550	−3.14	231.0642, 153.0180
Kaempferol-3-rutinoside	C_27_H_30_O_15_	13.35	[M+H]^+^	595.1655	595.1657	−0.34	449.1085, 287.0560
Kaempferol-3-O-glucosyl	C_21_H_20_O_11_	13.66	[M+H]^+^	449.1086	449.1078	1.78	287.0561, 127.0380
Kwanzoquinone E	C_15_H_10_O_6_	13.66	[M+H]^+^	287.0541	287.0550	−3,14	241.0475, 231.0642, 213.0549, 121.0299, 107.0482
Guajavarin	C_20_H_18_O_11_	13.74	[M+H]^+^	435.0920	435.0922	−0.46	303.0495, 287.0556, 195.0295, 155.0337, 137.0596, 121.0651
Kwanzoquinone F	C_21_H_20_O_11_	13.78	[M+H]^+^	449.1094	449.1078	3.56	287.0551, 259.0609, 257.0439, 201.0547, 169.0498, 139.0391, 123.0441, 121.0287
Isorhamnetin-3-glucopyranoside	C_22_H_22_O_12_	14.08	[M+H]^+^	479.1158	479.1184	−5.43	317.0662, 127.0390
Hesperidin	C_28_H_34_O_15_	14.30	[M+H]^+^	611.1964	611.1970	–0.98	327.1297, 303.0865, 273.0761, 181.0495, 165.0761, 125.0597
Kaempferol 3-*α*-arabinopyranoside	C_20_H_18_O_10_	14.47	[M+H]^+^	419.0972	419.0973	−0.24	287.0555, 195.0294, 155.0337, 139.0391, 127.0390, 111.0441
Huanghua anthraquinone	C_16_H_12_O_6_	15.13	[M+H]^+^	301.0706	301.0707	−0.33	285.0757, 273.0759, 245.0804, 183.0652, 167.0703, 155.0704, 139.0753, 109.0649, 107.0493
Chrysophanic acid	C_15_H_10_O_4_	15.91	[M+H]^+^	255.0652	255.0652	0.00	199.0753, 183.0803, 137.0595, 123.0441, 121.0647, 109.0648
3′-methoxy puerarin	C_22_H_22_O_10_	18.04	[M+H]^+^	447.1290	447.1280	0.89	285.0766
3*α*-acetyl-11-oxo-12-ursene-24-carboxylic acid	C_33_H_48_O_5_	19.03	[M+H]^+^	513.3583	513.3575	1.55	455.3509, 281.1744, 235.2056, 223.1695, 219.2110
11*α*-hydroxy-3-hexanoyl-*β*-boswellic acid	C_32_H_50_O_5_	19.92	[M+H]^+^	515.3731	515.3731	0.00	281.1749, 235.2052, 223.1693, 211.2060, 185.1537
Kwanzoquinone A	C_18_H_14_O_4_	21.17	[M+H]^+^	295.0976	295.0965	3.05	179.0706, 149.0600, 137.0602, 121.0652
Kwanzoquinone B	C_18_H_14_O_4_	21.17	[M+H]^+^	295.0976	295.0965	3.05	179.0706, 149.0600, 137.0602, 121.0652
*α*-boswellic acid	C_30_H_48_O_3_	22.44	[M+H]^+^	457.3676	457.3676	0.00	461.3972, 441.3719, 439.3560, 237.1485, 221.1535, 219.2106, 191.1789
*β*-boswellic acid	C_30_H_48_O_3_	22.44	[M+H]^+^	457.3676	457.3676	0.00	461.3972, 441.3719, 439.3560, 237.1485, 221.1535, 219.2106, 191.1789

**Table 2:** Main active constitutes of HCB.

No.	Name	Degree	Betweenness Centrality	Closeness Centrality
1	Quercetin	38	0.2635	0.6213
2	Kaempferol	22	0.0794	0.4740
3	Clionasterol	5	0.0102	0.3786
4	Guajavarin	5	0.0029	0.3786
5	Isoquercetin	5	0.0029	0.3786
6	Hyperoside	5	0.0029	0.3786
7	Adenosine	5	0.0077	0.3786
8	Kaempferol-3-rutinoside	4	0.0029	0.3742
9	Rutin	4	0.0029	0.3742
10	Chrysophanic acid	2	0.0020	0.3657
11	Gallic acid	2	4.4434	0.3657
12	Kwanzoquinone G	2	0.0012	0.3657
13	Rhein	2	0.0012	0.3657

## Data Availability

The original contributions presented in this study are included in the article/Supplementary Material. Further inquiries can be directed to the corresponding author Yuqin Yang.

## Supplementary Materials

The following supporting information can be downloaded at: https://www.mdpi.com/article/10.3390/ph17121704/s1. Figure S1: Product ion spectra of (A) 3′-methoxy puerarin, (B) hesperidin, (C) catechin, (D) quercetin-3,7-2-O-glucose, (E) guajavarin, (F) quercetin 3-O-rutinoside-7-O-glucoside, (G) hyperoside, (H) rutin; Figure S2: Product ion spectra of (A) kaempferol, (B) kaempferol 3-α-arabinopyranoside, (C) kaempferol-3-O-glucosyl, (D) kaempferol-3-O-rutinoside, (E) hemerocallone, (F) isoquercetin, (G) isorhamnetin-3-glucopyranoside; Figure S3: Product ion spectra of (A) chrysophanic acid, (B) kwanzoquinone E, (C) 2-hydroxychrysophanol, (D) kwanzoquinone G, (E) huanghua anthraquinone, (F) kwanzoquinone B, (G) aloe emodin, (H) kwanzoquinone A; Figure S4: Product ion spectra of chrysoobtusin; Figure S5: Product ion spectra of (A) β-boswellic acid, (B) 11α-hydroxy-3-hexanoyl-β-boswellic acid, (C) 3α-acetyl-11-oxo-12-ursene-24-carboxylic acid; Figure S6: Product ion spectra of (A) kwansonine B, (B) kwansonine C, (C) longitubanine A, (D) longitubanine B, (E) 1′,2′,3′,4′-tetrahydro, (F) adenosine, (G) fulvanine A, (H) oxypinnatanine; Figure S7: Product ion spectra of (A) oxypinnatanine, (B) pinnatanine; Figure S8: Product ion spectra of (A) 3-O-feruloylquinic acid, (B) 3-O-p-coumaroylquinic acid, (C) 4-O-p-coumaroylquinic acid, (D) 4-O-caffeoyl-quinic acid, (E) 4-O-caffeoylshikimic acid, (F) quinic acid, (G) methyl chlorogenate, (H) neochlorogenic acid; Figure S9: Product ion spectra of cryptochlorogenic acid; Figure S10: Product ion spectra of (A) vanillic acid, (B) ferulic acid, (C) syringic acid, (D) gallic acid; Figure S11: Product ion spectra of (A) icariside D2, (B) salidroside; Figure S12: HCB regulated NF-κB signaling pathway and ROS (A) Representative protein bands of p65 and p-p65 in hippocampal. (B) Statistical graphs of relative protein expression of ratio of p-65/GAPDH, p-p65/GAPDH Data are presented as mean ± SEM, **** *p* < 0.0001.(C) Representative protein bands of p65 and p-p65 in SY5Y. (D) Statistical graphs of relative protein expression of ratio of p-65/GAPDH, p-p65/GAPDH Data are presented as mean ± SEM, ** *p* < 0.01, **** *p* < 0.0001.(E) HCB can alleviate ROS production induced by LPS at the cellular level.(F)CCK8 results for LPS. Table S1: Main effective target of HCB; Table S2: The affinity energy of component with PIK3R1; Table S3: The stressors of CUMS protocol, which was randomly assigned over a week period and repeated throughout 6 weeks experiment; Table S4: Information on the constituents identified in dried flowers of HCB in positive ionization mode.

## Abbreviations

5-HT, 5-hydroxytryptamine; Akt, protein kinase B; BDNF, brain-derived neurotrophic factor; CUMS, chronic unpredictable mild stress; CREB, cAMP-response element binding protein; DA, dopamine; DEGs, differentially expressed genes; FST, forced swimming test; GAPDH, glyceraldehyde phosphate dehydrogenase; GO, Gene Ontology; KEGG, Kyoto Encyclopedia of Genes and Genomes; LSD, least square differences; OFT, open filed test; p-Akt, phospho-protein kinase B; p-CREB, phosphor-cAMP-response element binding protein; p-PI3K, phospho-phosphoinositide-3-kinase; PI3K, phosphoinositide-3-kinase; PPI, protein–protein interactions; RDA, retro-Diels–Alder; SPT, sucrose preference test; TCM, traditional Chinese medicine; TICC, total ion currant chromatogram; UHPLC-Q-Orbitrap HRMS, ultra-high-performance liquid chromatography coupled to quadrupole-orbitrap high-resolution mass spectrometry.
