# Supplementary material for: Revealing the Mechanism of Hemerocallis citrina Baroni in Depression Treatment Through Integrated Network Pharmacology and Transcriptomic Analysis
Source: Pharmaceuticals (Basel). 2024 Dec 17;17(12):1704. doi: 10.3390/ph17121704 (PMC11677347; doi:10.3390/ph17121704)
Supplement: Supplementary file 1 [file pharmaceuticals-17-01704-s001.zip › pharmaceuticals-3335169-supplementary.pdf]

Experimental Section

UHPLC-Q-Orbitrap HRMS Analysis.

Chemical constituents in HCB were investigated by using the UHPLC-Q-Orbitrap-HRMS. The mobile phase was composed of water/0.1% formic acid (A) and acetonitrile (B). The elution conditions were set as follows: 0–10 min, 4.0–20% B; 10–15 min, 20–40% B; 15–17 min, 40-70% B; 17–20 min, 70-90% B; 20–25 min, 90% B; 25–26 min, 90-4.0% B; 26–30 min, 4.0% B. Full-scan data within the range of *m/z* (mass-to-charge ratio) 100–1500 were acquired.

Network Pharmacology Analysis.

The compounds in HCB would be identified. Before forecasting, the "Canonical SMILES" of each compound should be acquired from PubChem (https://pubchem.ncbi.nlm.nih.gov/). Targets of compounds in HCB were predicated with their "Canonical SMILES" on Swiss Target Prediction database (http://swisstargetprediction.ch/). To analyze the therapeutic targets of depression in scientific texts deposited in MEDLINE database, a data-mining analysis of scientific literature was performed using GenClip3 (http://ci.smu.edu.cn/genclip3/analysis.php) web-service. The venn diagrams were produced by using the online software Venny.2.1 to show the number of targets linked to depression and identified compounds. Then the intersection was exported to R software. Then the software Cytoscape 3.9.1 was used to construct the "compounds-targets-diseases" network. Protein-protein interactions (PPI) in human genome were extracted from version 11.5 of STRING (https://STRING-db.org/), a weighted interaction database containing physical and functional interactions that are integrated from multiple data sources. Based on the above analyses, the intersecting targets of HCB and anti-depression were used to build the PPI network. Subsequently, the overlap targets were submitted to STRING tool to acquire PPI relationships with the species limited to "Homo sapiens." In order to construct a PPI network with high confidence edges, we filtered the STRING with threshold 0.7. Only interactions with weight above the threshold were selected for the newly constructed PPI network. Finally, Cytoscape 3.9.1 was used to visualize the PPI network. The three topological properties, "degree", "betweenness", and "closeness", were calculated to screen the putative targets for topological importance. Gene Ontology (GO) and Kyoto Encyclopedia of Genes and Genomes (KEGG) pathway enrichment analysis. To clarify the pathways that are involved in putative HCB targets, DAVID 6.8 (https://david.ncifcrf.gov) was used to perform the GO and KEGG enrichment. In the study, first step, a gene list was entered into the search box, subsequently selected identifier "OFFICIAL GENE SYMBOL" and chose list type "Gene List", then submitted the list. The second step, "Homo sapiens" was selected to limit annotations and selected "List 1". The third step, the background of "Homo sapiens" was selected. The last step, the "Functional Annotation Chart" was selected to obtain GO ("GOTERM-BP- DIRECT") and KEGG ("KEGG-

PATHWAY") Pathway Analysis results. Finally, the functional categories were identified and ranked by p-values, and those GO terms and KEGG pathways with p-value≤0.001 were recognized as significant.

Behavioral Test.

The mice were deprived of food and water duration 24 h before SPT. Two bottles filled with distilled water and 1% sucrose solution which were weighted and marked in advance were given to the mice for 1 h. The consumption of sucrose solution and distilled water was recorded after 1 h. Then, two bottles were removed simultaneously and measured. The sucrose preference was calculated by: the amount of sugar water consumed/the total amount consumed. OFT provided simultaneous measures of movement, exploration, and anxiety. An open box (50 cm × 50 cm × 50 cm) was placed in a quiet room, the black floor was divided into 16 equal-sized squares and four side walls, and a computer above the middle of the box was connected to a video camera. The mice were placed in the center of the experimental device and explored freely for 5 min. After each experiment, the feces were cleaned with 75% ethanol and the experimental apparatus was wiped. The distance moved, cumulative duration in the center and not moving time were tracked and measured by the software EthoVision XT 9. FST was performed in a cylinder with a height of 30 cm × a diameter of 17 cm, and the mice were placed in it. The cylinder contained 25 ± 2 °C of water which a depth was about 15 cm, so that the mice could not support their bodies with feet touching the bottom. FST was performed for 4 min after 2 min adaption, and the mice were trained to learn swimming before FST 24 h. The immobility time during the 4 min was recorded.

Nissl staining. For Nissl staining, brain sections were dewaxed with xylene, hydrated with 75% - 90% graded alcohol, stained with Nissl solution for 10 min, washed with distilled water, dehydrated with anhydrous ethanol, transparentized with xylene and fixed with neutral balm. Whole tissues in images were observed and photographed with digital slide scanner under 10x microscopy to identify Nissl bodies, select areas to be observed and take 40x pictures to count Nissl bodies.

ELISA Test.

50 μL of standards and samples were added into the appropriate wells, and blank well was not added anything. Then 100μl of enzyme conjugate was added to standard wells and sample wells except the blank well, covered with an adhesive strip and incubated for 60 min at 37 °C. After washing the microtiter plate 4 times, substrate A and B were added into each well and incubated for 15 min at 37 °C with protecting from light. Finally, the Optical Density (O.D.) was read at 450 nm using a microtiter plate reader within 15 min after adding 50μl stop solution into each well.

Western Bolt.

Expression levels of p-PI3K, PI3K, p-Akt, Akt, p-CREB, and CREB were measured by western blot analysis. The total protein of hippocampal tissues was extracted using RIPA Lysis buffer. After mixed the loading buffer and heated at 100 °C for 5 min, the proteins were separated on the 4%-12% SDS-PAGE gels at 30 μg and transformed onto a PVDF membrane. The PVDF membranes were blocked using the 5% non-fat dry milk in 0.05% TBST and then were incubated at 4 °C overnight with primary antibodies. The PVDF membranes were washed with TBST for 5 min three times, followed by incubation with secondary antibody conjugated to horseradish peroxidase for 1 h at room temperature. The PVDF membranes were washed in the same way, and protein bands were detected by ECL hypersensitive luminescent liquid.

RNA Extraction from Mice Hippocampus.

Total RNA was extracted from the tissue using TRIzol® Reagent according the manufacturer's instructions (Invitrogen) and genomic DNA was removed using DNase I (TaKara). RNA quality was determined by 2100 Bioanalyser (Agilent) and quantified using the ND-2000 (NanoDrop Technologies). Only high-quality RNA sample (OD260/280=1.8~2.2, OD260/230≥2.0, RIN≥6.5, 28S:18S≥1.0, >1μg) was used to construct sequencing library.

Library Preparation, and Illumina Hiseq xten/Nova seq 6000 Sequencing.

RNA-seq transcriptome librariy was prepared following TruSeqTM RNA sample preparation Kit from Illumina (San Diego, CA) using 1μg of total RNA. Shortly, messenger RNA was isolated according to polyA selection method by oligo (dT) beads and then fragmented by fragmentation buffer firstly. Secondly double-stranded cDNA was synthesized using a SuperScript double-stranded cDNA synthesis kit (Invitrogen, CA) with random hexamer primers (Illumina). Then the synthesized cDNA was subjected to end-repair, phosphorylation and 'A' base addition according to Illumina's library construction protocol. Libraries were size selected for cDNA target fragments of 300 bp on 2% Low Range Ultra Agarose followed by PCR amplified using Phusion DNA polymerase (NEB) for 15 PCR cycles. After quantified by TBS380, paired-end RNA-seq sequencing library was sequenced with the Illumina HiSeq xten/NovaSeq 6000 sequencer (2 × 150bp read length).

Read Mapping.

The raw paired end reads were trimmed and quality controlled by SeqPrep (https://github.com/jstjohn/SeqPrep) and Sickle (https://github.com/najoshi/sickle) with default parameters. Then clean reads were separately aligned to reference genome with orientation mode using HISAT2 (http://ccb.jhu.edu/software/hisat2/index.shtml) software. The mapped reads of each sample were assembled by StringTie (https://ccb.jhu.edu/software/stringtie/index.shtml?t=example) in a reference-based approach. Then, R studio was used for

downstream bioinformatics analysis. Differential expression gene was extracted with Limma package.

Differential Expression Analysis and Functional Enrichment.

To identify DEGs (differential expression genes) between two different samples, the expression level of each transcript was calculated according to the transcripts per million reads (TPM) method. RSEM (http://deweylab.biostat.wisc.edu/rsem/) was used to quantify gene abundances. Essentially, differential expression analysis was performed using the DESeq2/DEGseq/EdgeR with Q value ≤ 0.05, DEGs with |log2FC|>1 and Q value <= 0.05(DESeq2 or EdgeR) /Q value <= 0.001(DEGseq) were considered to be significantly different expressed genes). In addition, functional-enrichment analysis including GO and KEGG were performed to identify which DEGs were significantly enriched in GO terms and metabolic pathways at Bonferroni-corrected P-value ≤0.05 compared with the whole-transcriptome background. GO functional enrichment and KEGG pathway analysis were carried out by Goatools (https://github.com/tanghaibao/Goatools) and KOBAS (http://kobas.cbi.pku.edu.cn/home.do).

Table S1. Main effective target of HCB.

No.	Target name	Common name	Uniprot ID	Degree	Betweenness Centrality	Closeness Centraity
1	RAC-alpha serine/threonine-protein kinase	AKT1	P31749	31	0.4039	0.6811
2	Proto-oncogene tyrosine-protein kinase Src	SRC	P12931	23	0.1769	0.6025
3	Epidermal growth factor receptor	EGFR	P00533	19	0.0552	0.5340
4	Matrix metalloproteinase-9	MMP9	P14780	16	0.0304	0.5108
5	Glycogen synthase kinase-3 beta	GSK3B	P49841	15	0.0310	0.5222
6	Androgen receptor	AR	P10275	14	0.0321	0.4947
7	72 kDa type IV collagenase	MMP2	P08253	14	0.0176	0.4895
8	Insulin-like growth factor 1 receptor	IGF1R	P08069	13	0.0047	0.4845
9	Phosphatidylinositol 3-kinase regulatory subunit alpha	PIK3R1	P27986	12	0.0063	0.4845
10	Microtubule-associated protein tau	MAPT	P10636	11	0.0605	0.4947

Table S2. The affinity energy of component with PIK3R1.

Targets	PDB ID	Compounds	Affinity(kcal/mol)
PIK3R1	7myo	Gallic acid	-6.2
		Kaempferol-3-rutinoside	-9.9
		quercetin	-7.7
		Rhein	-8.8
		Rutin	-9.6
		Isoquercetin	-8.7
		Chrysophanic acid	-8.3
		Hyperoside	-9.7

Table S3. The stressors of CUMS protocol, which was randomly assigned over a week period and repeated throughout 6 weeks experiment.

	Monday	Tuesday	Wednesday	Thursday	Friday	Saturday	Sunday
Food and water deprivation	✓						
Ice water swimming		✓					
Heat stress			✓				
Restraint stress				✓			
Walking on the ice					✓		
Noise and flash						✓	
All night lighting							✓

Table S4. Information on the constituents identified in dried flowers of HCB in positive ionization mode.

**Constituents**	**Formula**	**Retention time (min)**	**Identity**	Precursor ion			Fragment ions (*m/z*)
				Experimental (*m/z*)	Theoretical (*m/z*)	Mass accuracy (△ppm)	
**Clionasterol**	**$C_{29}H_{50}O$**	**0.32**	**$[M+H]^+$**	415.3934	415.3933	–0.24	397.3834,179.1434,123.1168
Quinic acid	$C_7H_{12}O_6$	1.43	$[M+H]^+$	193.0708	193.0707	0.52	175.0600, 157.0494, 133.0495, 115.0389
Kwansonine A	$C_{16}H_{26}N_2O_{11}$	1.44	$[M+H]^+$	423.1609	423.1609	0.00	261.1081, 245.1131, 243.0973, 217.1178, 202.1068, 163.0712, 146.0447, 145.0607, 144.0658
Oxypinnatanine	$C_{10}H_{16}N_2O_6$	1.50	$[M+H]^+$	261.1083	261.1081	0.77	246.0971, 245.1132, 230.1023, 215.1021, 200.0911, 187.1077, 163.0712, 146.0447, 130.0498
Syringic acid	$C_9H_{10}O_5$	1.97	$[M+H]^+$	199.0602	199.0601	0.50	183.0652, 169.0494, 153.0541, 151.0389, 139.0388, 123.0439
Pinnatannine	$C_{10}H_{16}N_2O_5$	2.07	$[M+H]^+$	245.1134	245.1132	0.82	228.0856, 227.1021, 128.0704, 100.0759, 84.0449
Oxypinnatanine A	$C_{10}H_{16}N_2O_5$	2.36	$[M+H]^+$	245.1132	245.1132	0.00	230.1023, 228.0863, 215.1029, 201.1233, 198.0763, 186.1128, 156.1022, 144.0655, 116.0707
Longitubanine A	$C_{10}H_{16}N_2O_2$	2.40	$[M+H]^+$	245.1130	245.1132	-0.82	227.1026, 210.0765, 201.1238, 172.0966, 163.0716, 156.1020, 145.0607, 120.0655, 100.0757
**Constituents**	**Formula**	**Retention time (min)**	**Identity**	Precursor ion			Fragment ions (*m/z*)
				Experimental (*m/z*)	Theoretical (*m/z*)	Mass accuracy	

						(△ppm)	
**Adenosine**	**$C_{10}H_{13}N_5O_4$**	**2.67**	**$[M+H]^+$**	268.1040	268.1040	0.00	136.0620
Kwansonine B	$C_{16}H_{26}N_2O_{10}$	3.25	$[M+H]^+$	407.1659	407.1660	-0.25	391.1699, 325.1236, 245.1129, 229.1178, 163.0711, 147.0762
Longitubanine B	$C_{10}H_{16}N_2O_4$	3.28	$[M+H]^+$	229.1182	229.1183	-0.44	214.1070, 170.1172, 147.0764, 132.0655, 130.0499, 128.0704, 117.0545, 104.0704
Kwansonine C	$C_{16}H_{26}N_2O_{10}$	3.48	$[M+H]^+$	407.1660	407.1660	0.00	391.1700, 307.1130, 245.1131, 229.1180, 163.0712, 145.0608
Fuluanine A	$C_9H_{13}NO_5$	5.46	$[M+H]^+$	216.0869	216.0866	1.39	198.0763, 186.0760, 118.0499
Vanillic acid	$C_8H_8O_4$	5.49	$[M+H]^+$	169.0497	169.0495	1.18	153.0547, 139.0390, 125.0598, 123.0440, 109.0648
Chlorogenic acid	$C_{16}H_{18}O_9$	6.39	$[M+H]^+$	355.1024	355.1024	0.00	337.0901, 165.0542, 163.0390, 145.0283, 137.0595, 135.0442, 117.0336
Cryptochlorogenic acid	$C_{16}H_{18}O_9$	6.39	$[M+H]^+$	355.1024	355.1024	0.00	337.0901, 165.0542, 163.0390, 145.0283, 137.0595, 135.0442, 117.0336
Neochlorogenic acid	$C_{16}H_{18}O_9$	6.39	$[M+H]^+$	355.1024	355.1024	0.00	337.0901, 165.0542, 163.0390, 145.0283, 137.0595, 135.0442, 117.0336

**Constituents**	**Formula**	**Retention time (min)**	**Identity**	Precursor ion			Fragment ions (*m/z*)
				Experimental (*m/z*)	Theoretical (*m/z*)	Mass accuracy (△ppm)	
**Salidroside**	**$C_{14}H_{20}O_7$**	**6.88**	**$[M+H]^+$**	301.1280	301.1282	-0.66	285.1331, 153.0910, 149.0962, 139.0754, 123.0805, 107.0856

**Constituents**	**Formula**	**Retention time (min)**	**Identity**	Precursor ion			Fragment ions (*m/z*)
				Experimental (*m/z*)	Theoretical (*m/z*)	Mass accuracy (△ppm)	
Icariside D2	$C_{14}H_{20}O_7$	6.89	$[M+H]^+$	301.1282	301.1281	0.00	283.1176, 265.1069, 235.0963, 139.0755, 107.0857
7-hydroxycoumarin	$C_9H_6O_3$	6.93	$[M+H]^+$	163.0386	163.0390	-2.45	147.0436, 145.0284, 135.0445,
1’,2’,3’,4’-tetraphydro-5'-deoxypinnatanine	$C_{10}H_{20}N_2O_4$	7.42	$[M+H]^+$	233.1503	233.1496	3.00	216.1234, 215.1384, 146.0450, 84.0442, 73.0283
3-O-*p*-coumaroylquinic acid	$C_{16}H_{18}O_8$	7.93	$[M+H]^+$	339.1075	339.1064	0.29	323.1125, 247.0817, 193.0711, 175.0604, 165.0546, 157.0496, 143.0708, 139.0390, 121.0650
4-O-*p*-coumaroylquinic acid	$C_{16}H_{18}O_8$	7.93	$[M+H]^+$	339.1075	339.1064	0.29	247.0806, 193.0709, 175.0601, 165.0545, 157.0495, 147.0443, 139.0389, 121.0648, 101.0598
Quercetin-3,7-2-O-glucose	$C_{27}H_{30}O_{17}$	8.20	$[M+H]^+$	627.1568	627.1556	1.91	465.1039, 303.0493
Isoquercetin	$C_{21}H_{20}O_{12}$	8.34	$[M+H]^+$	465.1031	465.1028	0.65	303.0507, 127.0398
Quercetin 3-O-rutinoside-7-glucoside	$C_{33}H_{40}O_{21}$	8.59	$[M+H]^+$	773.2130	773.2135	0.65	627.1515, 611.1633, 465.1054, 303.0501
4-O-caffeoyl-quinic acid	$C_{16}H_{18}O_9$	8.61	$[M+H]^+$	355.1023	355.1024	-0.28	339.1080, 337.0914, 293.1022, 193.0712, 181.0497, 175.0603, 163.0390, 145.0287, 113.0596
**Methyl chlorogenate**	$\mathbf{C_{17}H_{20}O_9}$	**8.75**	$\mathbf{[M+H]^+}$	369.1180	369.1180	0.00	355.1023, 339.1080, 195.0653, 175.0604, 177.0545, 163.0385, 157.0497, 131.0706, 121.0650

3-O-feruloylquinic acid	$C_{17}H_{20}O_9$	8.75	$[M+H]^+$	433.1133	433.1129	0.92	271.0601, 153.0181, 127.0389
Gallic acid	$C_7H_6O_5$	9.21	$[M+H]^+$	171.0286	171.0288	-1.17	153.0179, 137.0237, 127.0389, 109.0281
Hemerocallone	$C_{18}H_{14}O_6$	9.87	$[M+H]^+$	327.0871	327.0863	2.44	165.0549, 163.0752, 137.0601, 127.0391
Puerarin	$C_{21}H_{20}O_9$	10.08	$[M+H]^+$	417.1176	417.1180	-0.96	255.0647, 165.0544, 163.0392, 149.0599, 139.0388, 123.0442
2-hydroxychrysophanol	$C_{15}H_{10}O_5$	10.24	$[M+H]^+$	271.0601	271.0601	0.00	243.0648, 215.0704, 153.0548, 135.0442, 125.0599, 109.0648
Aloe emodin	$C_{15}H_{10}O_5$	10.32	$[M+H]^+$	271.0602	271.0601	0.37	243.0658, 137.0596, 123.0441, 107.0490
Kwanzoquinone G	$C_{16}H_{10}O_6$	10.34	$[M+H]^+$	299.0550	299.0550	0.00	271.0601, 243.0648, 165.0548, 147.0441, 137.0596
4-O-caffeoylshikimic acid	$C_{16}H_{16}O_8$	10.60	$[M+H]^+$	337.0912	337.0918	-1.78	319.0812, 229.0708, 185.0812, 181.0496, 174.0530, 159.0657, 149.0956, 131.0705, 111.0440
Ferulic acid	$C_{10}H_{10}O_4$	10.62	$[M+H]^+$	195.0661	195.0652	4.69	177.0554, 149.0590, 125.0598, 95.0496, 79.0540
**Constituents**	**Formula**	**Retention time (min)**	**Identity**	Precursor ion			Fragment ions (*m/z*)
				Experimental (*m/z*)	Theoretical (*m/z*)	Mass accuracy (△ppm)	
**Phenethyl-*β*-D-glu**	**$C_{14}H_{20}O_6$**	**11.48**	**$[M+H]^+$**	285.1329	285.1333	-1.40	249.1117, 181.1222, 149.0962, 147.0806, 123.0805, 105.0699
Catechin	$C_{15}H_{14}O_6$	12.35	$[M+H]^+$	291.0858	291.0863	–1.72	183.0653, 169.0860, 167.0704, 153.0909, 137.0962, 109.0649

Constituents	Formula	Retention time (min)	Identity	Precursor ion Experimental (*m/z*)	Theoretical (*m/z*)	Mass accuracy (△ppm)	Fragment ions (*m/z*)
Chrysoobtusin	$C_{17}H_{14}O_5$	12.61	$[M+H]^+$	299.0913	299.0914	-0.33	271.0967, 181.0859, 151.0756, 123.0440, 121.0649
Quercetin	$C_{15}H_{10}O_7$	12.68	$[M+H]^+$	303.0499	303.0499	0.00	287.0547, 179.0336, 155.0338, 139.0388, 123.0439, 105.0699
Rutin	$C_{27}H_{30}O_{16}$	12.71	$[M+H]^+$	611.1627	611.1607	3.27	465.1049, 449.1090, 303.0499
Rhein	$C_{15}H_8O_6$	12.72	$[M+H]^+$	285.0394	285.0394	0.00	257.0438, 229.0501, 167.0337, 151.0387, 139.0390, 123.0441
Hyperoside	$C_{21}H_{20}O_{12}$	13.21	$[M+H]^+$	465.1021	465.1028	-1.51	303.0496
Kaempferol	$C_{15}H_{10}O_6$	13.66	$[M+H]^+$	287.0541	287.0550	-3.14	231.0642, 153.0180
Kaempferol-3-rutinoside	$C_{27}H_{30}O_{15}$	13.35	$[M+H]^+$	595.1655	595.1657	-0.34	449.1085, 287.0560
Kaempferol-3-O-glucosyl	$C_{21}H_{20}O_{11}$	13.66	$[M+H]^+$	449.1086	449.1078	1.78	287.0561, 127.0380
Kwanzoquinone E	$C_{15}H_{10}O_6$	13.66	$[M+H]^+$	287.0541	287.0550	-3,14	241.0475, 231.0642, 213.0549, 121.0299, 107.0482
Guajavarin	$C_{20}H_{18}O_{11}$	13.74	$[M+H]^+$	435.0920	435.0922	-0.46	303.0495, 287.0556, 195.0295, 155.0337, 137.0596, 121.0651
**Kwanzoquinone F**	$\mathbf{C_{21}H_{20}O_{11}}$	**13.78**	$\mathbf{[M+H]^+}$	449.1094	449.1078	3.56	287.0551, 259.0609, 257.0439, 201.0547, 169.0498, 139.0391, 123.0441, 121.0287
Isorhamnetin-3-glucopyranoside	$C_{22}H_{22}O_{12}$	14.08	$[M+H]^+$	479.1158	479.1184	-5.43	317.0662, 127.0390
Hesperidin	$C_{28}H_{34}O_{15}$	14.30	$[M+H]^+$	611.1964	611.1970	–0.98	327.1297, 303.0865, 273.0761, 181.0495, 165.0761, 125.0597

Kaempferol 3-*α*-arabinopyranoside	$C_{20}H_{18}O_{10}$	14.47	$[M+H]^+$	419.0972	419.0973	-0.24	287.0555, 195.0294, 155.0337, 139.0391, 127.0390, 111.0441
Huanghua anthraquinone	$C_{16}H_{12}O_6$	15.13	$[M+H]^+$	301.0706	301.0707	-0.33	285.0757, 273.0759, 245.0804, 183.0652, 167.0703, 155.0704, 139.0753, 109.0649, 107.0493
Chrysophanic acid	$C_{15}H_{10}O_4$	15.91	$[M+H]^+$	255.0652	255.0652	0.00	199.0753, 183.0803, 137.0595, 123.0441, 121.0647, 109.0648
3'-methoxy puerarin	$C_{22}H_{22}O_{10}$	18.04	$[M+H]^+$	447.1290	447.1280	0.89	285.0766
3*α*-acetyl-11-oxo-12-ursene-24-carboxylic acid	$C_{33}H_{48}O_5$	19.03	$[M+H]^+$	513.3583	513.3575	1.55	455.3509, 281.1744, 235.2056, 223.1695, 219.2110
11*α*-hydroxy-3-hexanoyl-*β*-boswellic acid	$C_{32}H_{50}O_5$	19.92	$[M+H]^+$	515.3731	515.3731	0.00	281.1749, 235.2052, 223.1693, 211.2060, 185.1537
Kwanzoquinone A	$C_{18}H_{14}O_4$	21.17	$[M+H]^+$	295.0976	295.0965	3.05	179.0706, 149.0600, 137.0602, 121.0652

**Constituents**	**Formula**	**Retention time (min)**	**Identity**	Precursor ion			Fragment ions (*m/z*)
				Experimental (*m/z*)	Theoretical (*m/z*)	Mass accuracy (△ppm)	
**Kwanzoquinone B**	$\mathbf{C_{18}H_{14}O_4}$	**21.17**	$\mathbf{[M+H]^+}$	295.0976	295.0965	3.05	179.0706, 149.0600, 137.0602, 121.0652
α-boswellic acid	$C_{30}H_{48}O_3$	22.44	$[M+H]^+$	457.3676	457.3676	0.00	461.3972, 441.3719, 439.3560, 237.1485, 221.1535, 219.2106, 191.1789
β-boswellic acid	$C_{30}H_{48}O_3$	22.44	$[M+H]^+$	457.3676	457.3676	0.00	461.3972, 441.3719, 439.3560, 237.1485, 221.1535, 219.2106, 191.1789

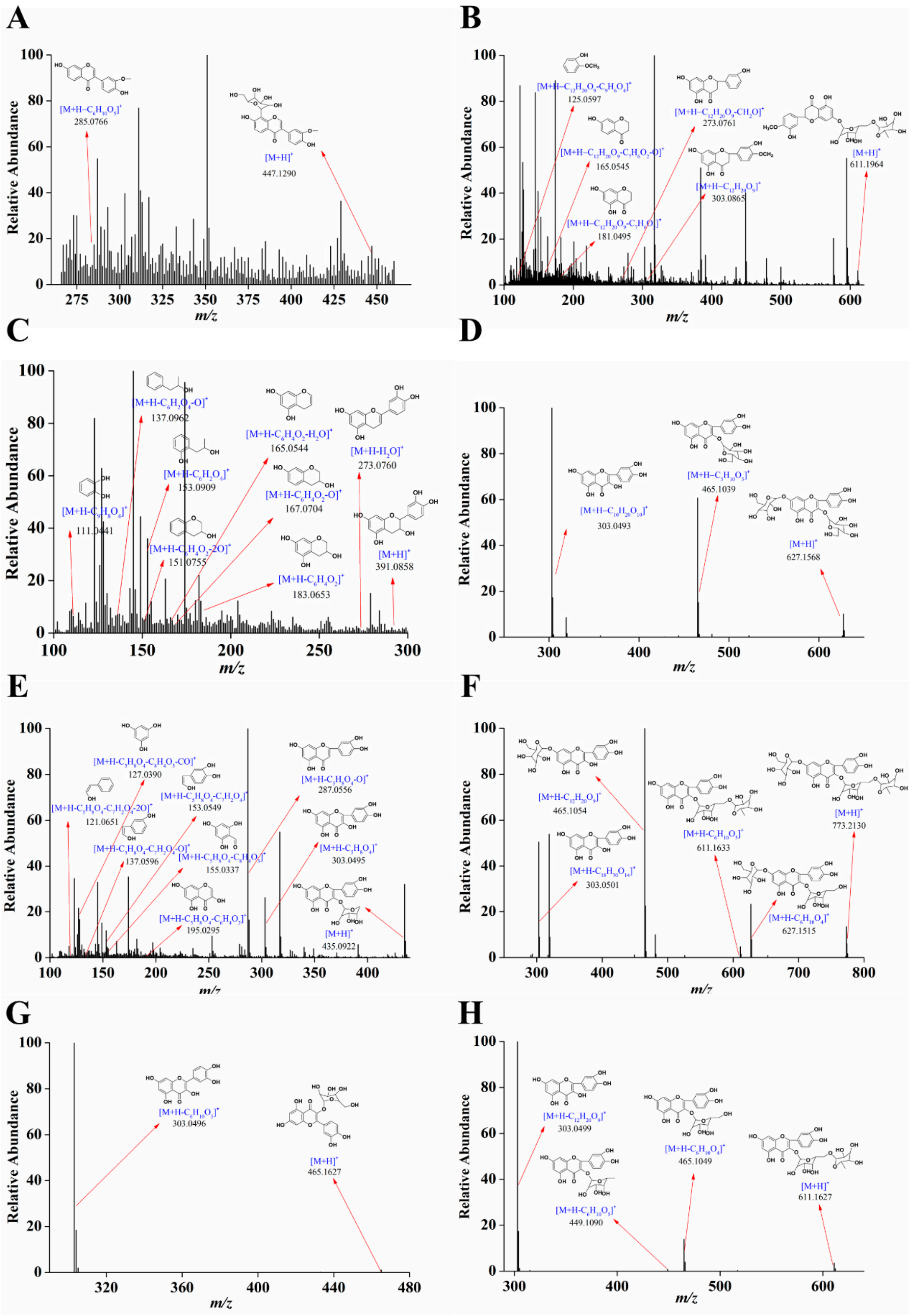

Figure S1. Product ion spectra of (A) 3'-methoxy puerarin, (B) hesperidin, (C) catechin, (D) quercetin-3,7-2-O-glucose, (E) guajavarin, (F) quercetin 3-O-rutinoside-7-O-glucoside, (G) hyperoside, (H) rutin.

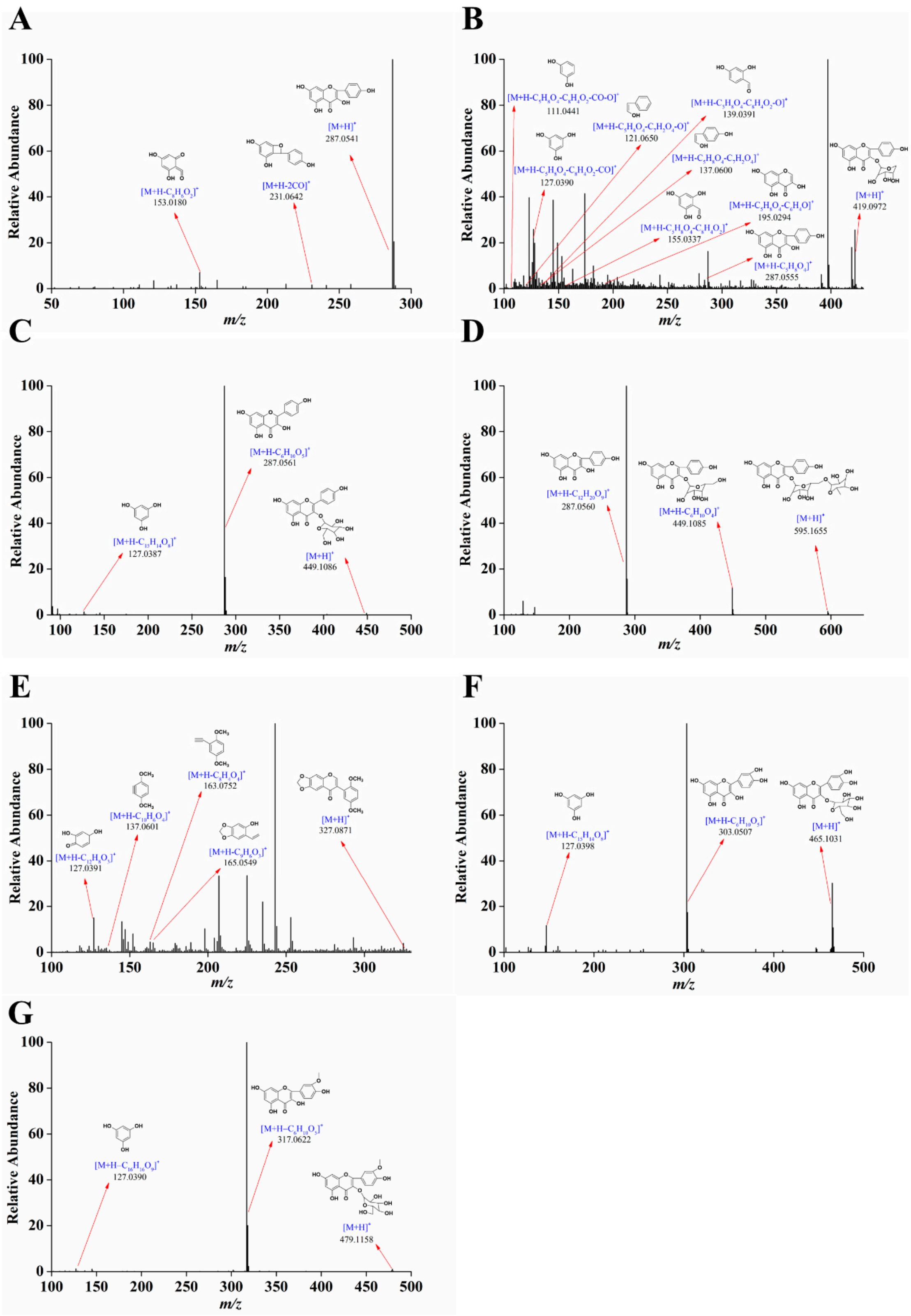

Figure S2. Product ion spectra of (A) kaempferol, (B) kaempferol 3-α-arabinopyranoside, (C) kaempferol-3-O-glucosyl, (D) kaempferol-3-O-rutinoside, (E) hemerocallone, (F) isoquercetin, (G) isorhamnetin-3-glucopyranoside.

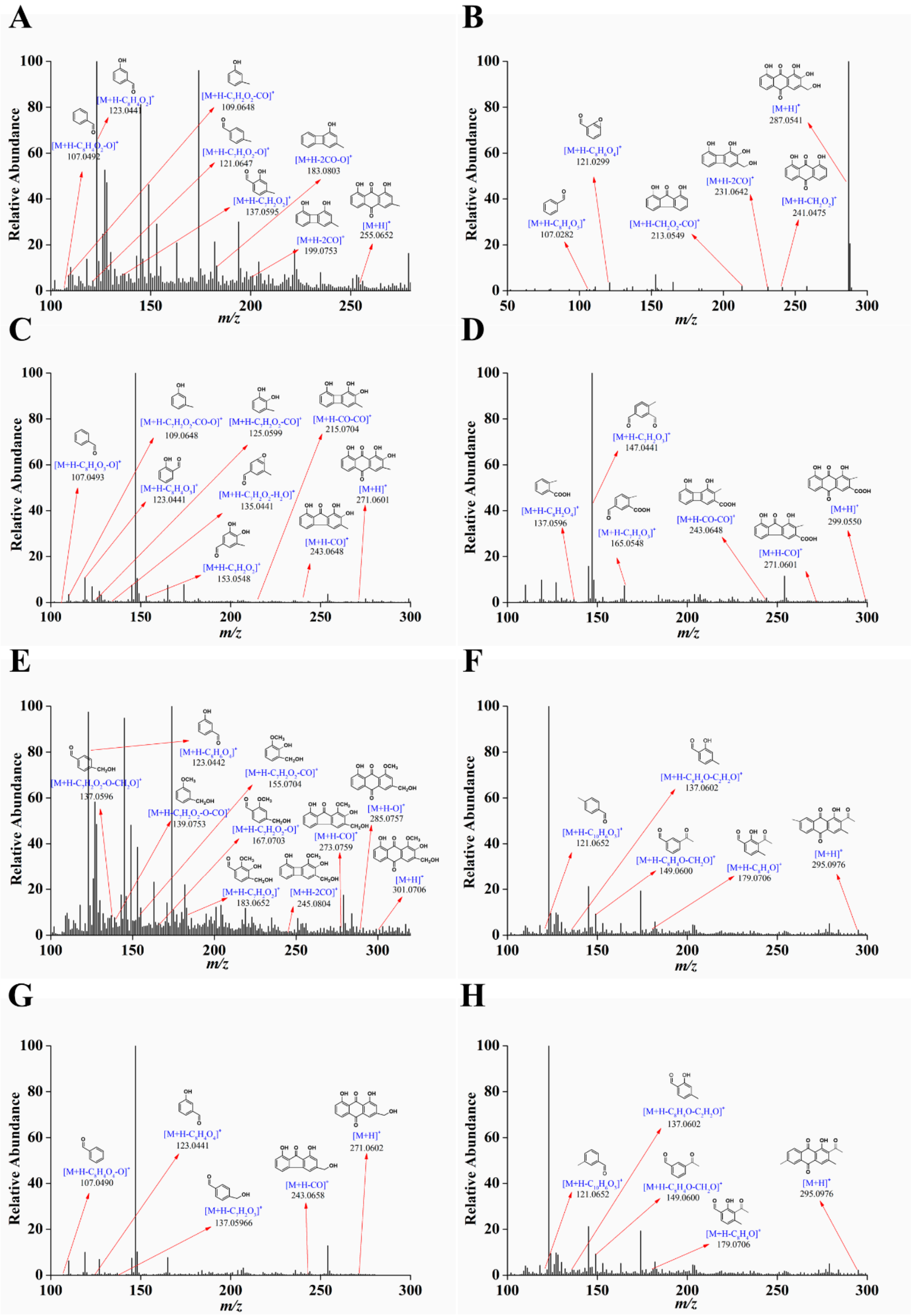

Figure S3. Product ion spectra of (A) chrysophanic acid, (B) kwanzoquinone E, (C) 2-hydroxychrysophanol, (D) kwanzoquinone G, (E) huanghua anthraquinone, (F) kwanzoquinone B, (G) aloe emodin, (H) kwanzoquinone A.

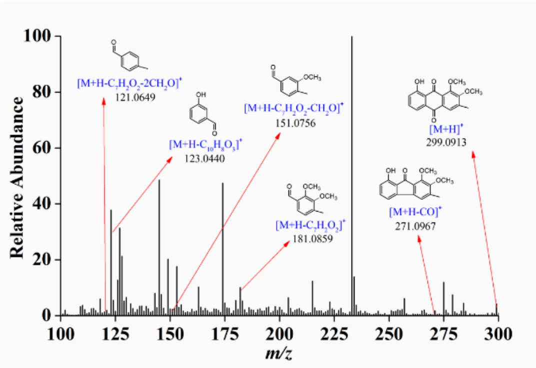

Figure S4. Product ion spectra of chrysoobtusin.

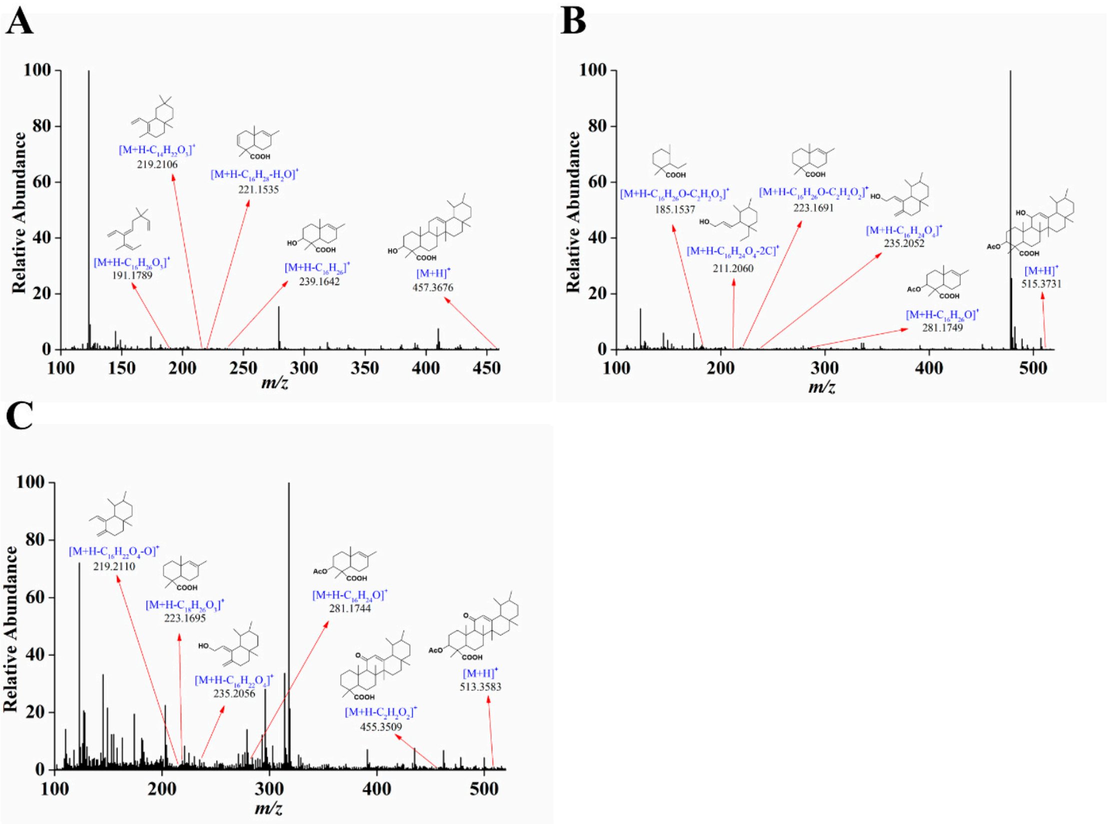

Figure S5. Product ion spectra of (A) β-boswellic acid, (B) 11α-hydroxy-3-hexanoyl-β-boswellic acid, (C) 3α-acetyl-11-oxo-12-ursene-24-carboxylic acid.

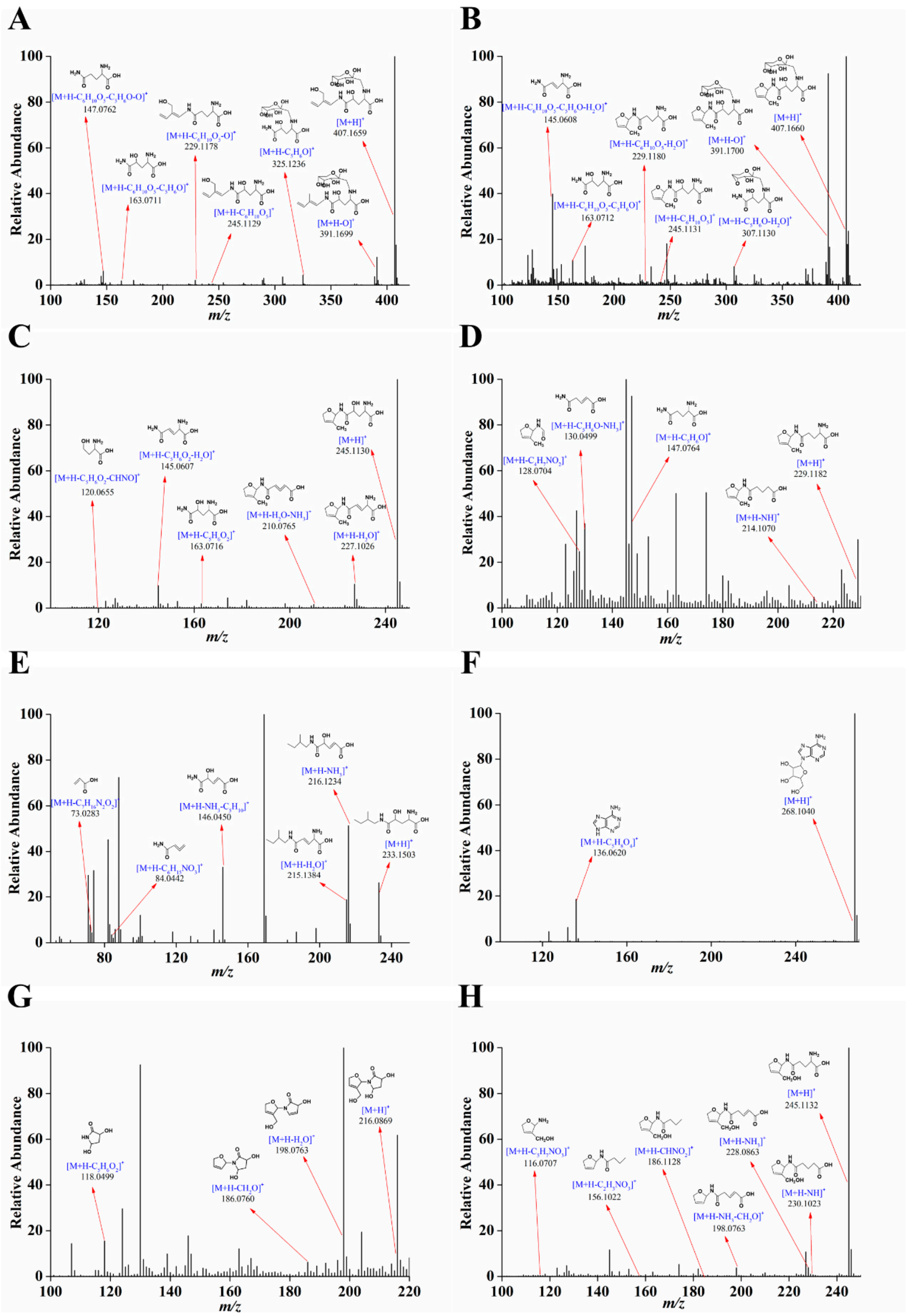

Figure S6. Product ion spectra of (A) kwansonine B, (B) kwansonine C, (C) longitubanine A, (D) longitubanine B, (E) 1',2',3',4'-tetrahydro, (F) adenosine, (G) fulvanine A, (H) oxypinnatanine A.

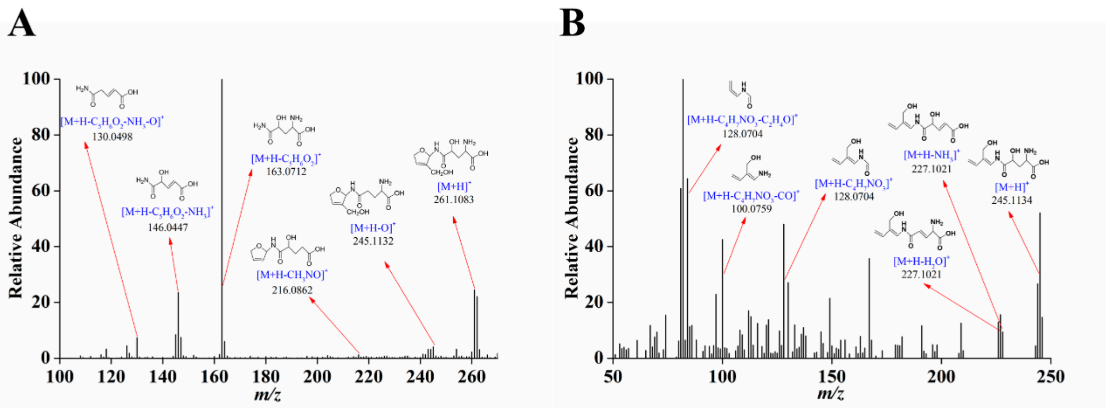

Figure S7. Product ion spectra of (A) oxypinnatanine, (B) pinnatanine.

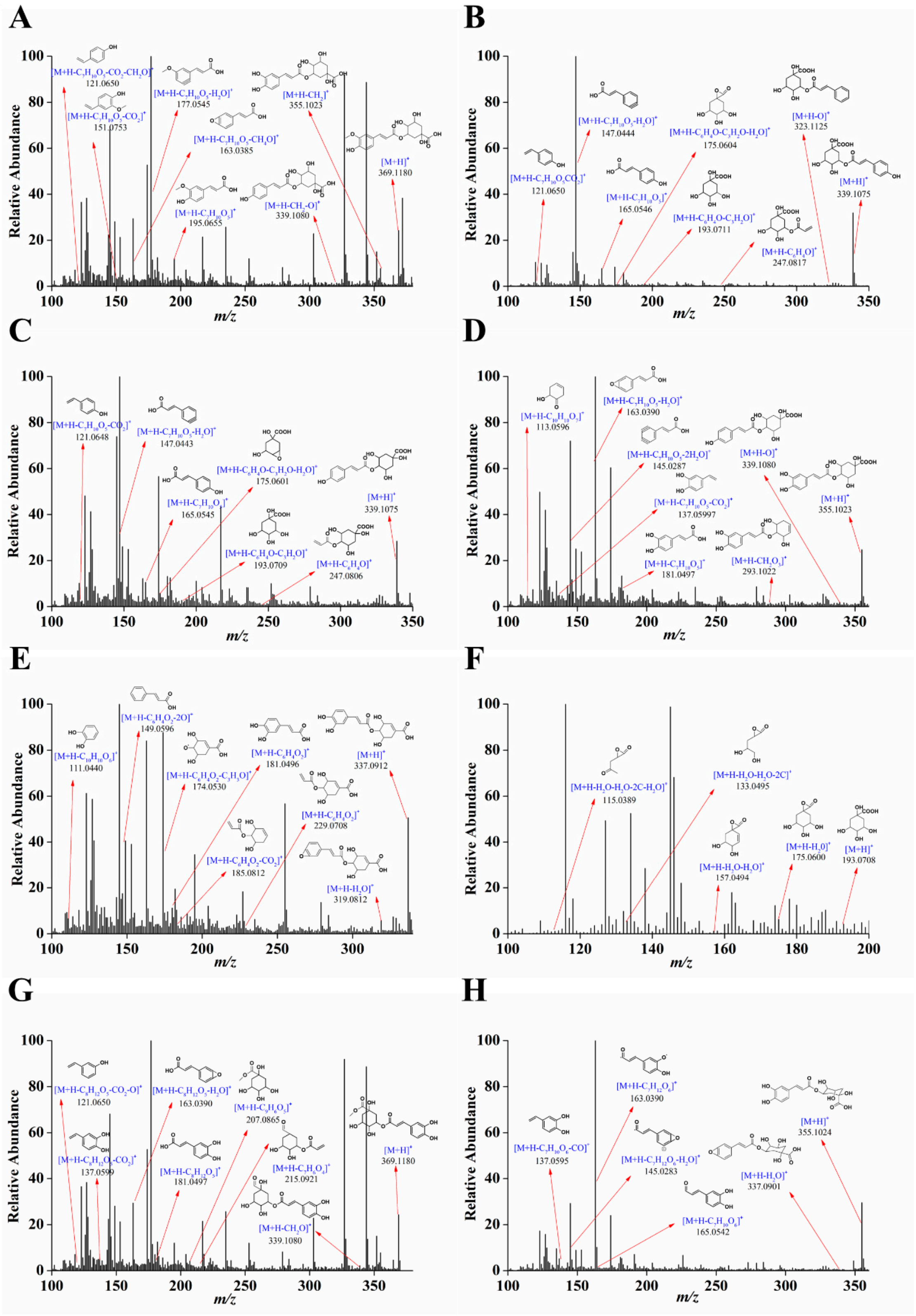

Figure S8. Product ion spectra of (A) 3-O-feruloylquinic acid, (B) 3-O-p-coumaroylquinic acid, (C) 4-O-p-coumaroylquinic acid, (D) 4-O-caffeoyl-quinic acid, (E) 4-O-caffeoylshikimic acid, (F) quinic acid, (G) methyl chlorogenate, (H) neochlorogenic acid.

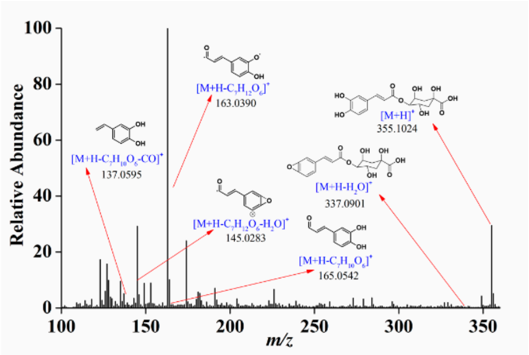

Figure S9. Product ion spectra of cryptochlorogenic acid.

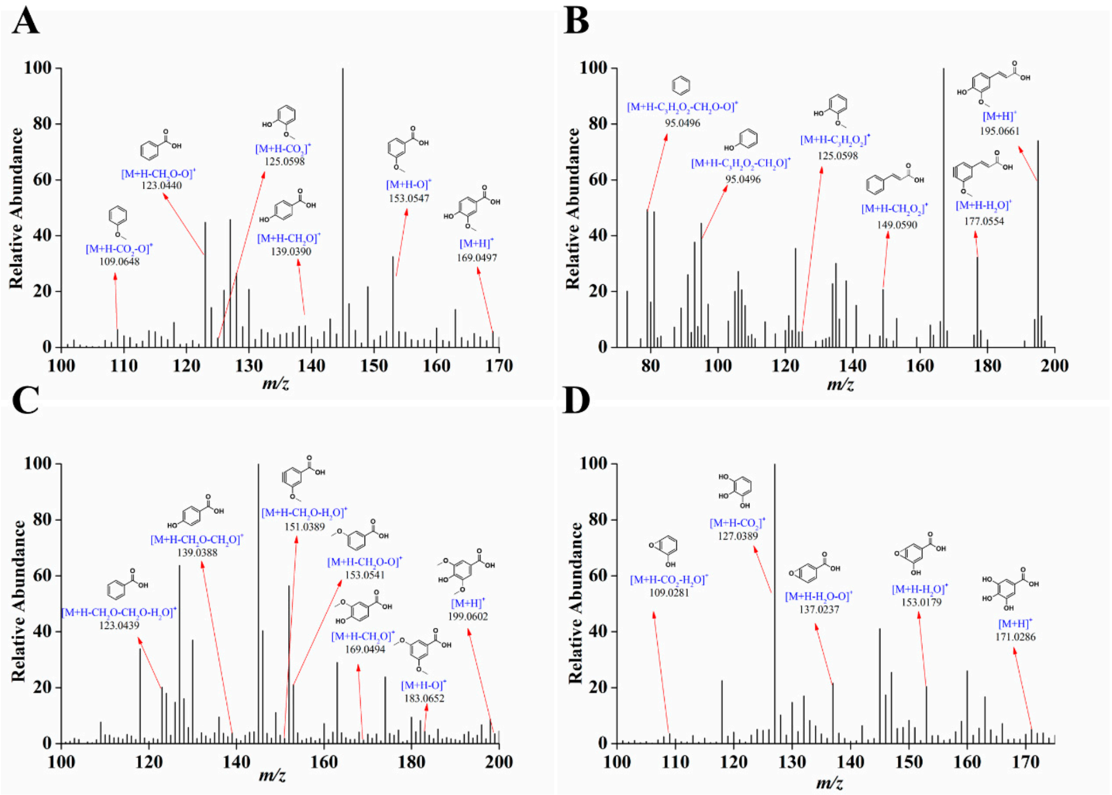

Figure S10. Product ion spectra of (A) vanillic acid, (B) ferulic acid, (C) syringic acid, (D) gallic acid.

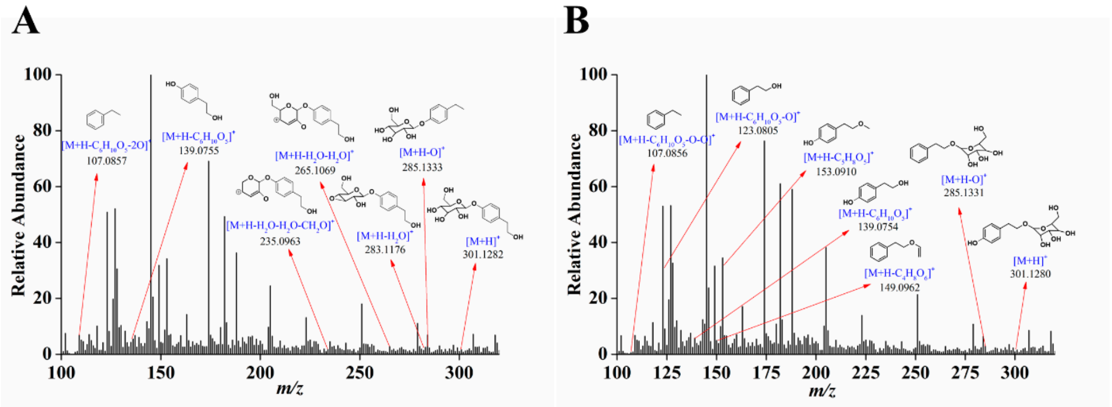

Figure S11. Product ion spectra of (A) icariside D2, (B) salidroside.

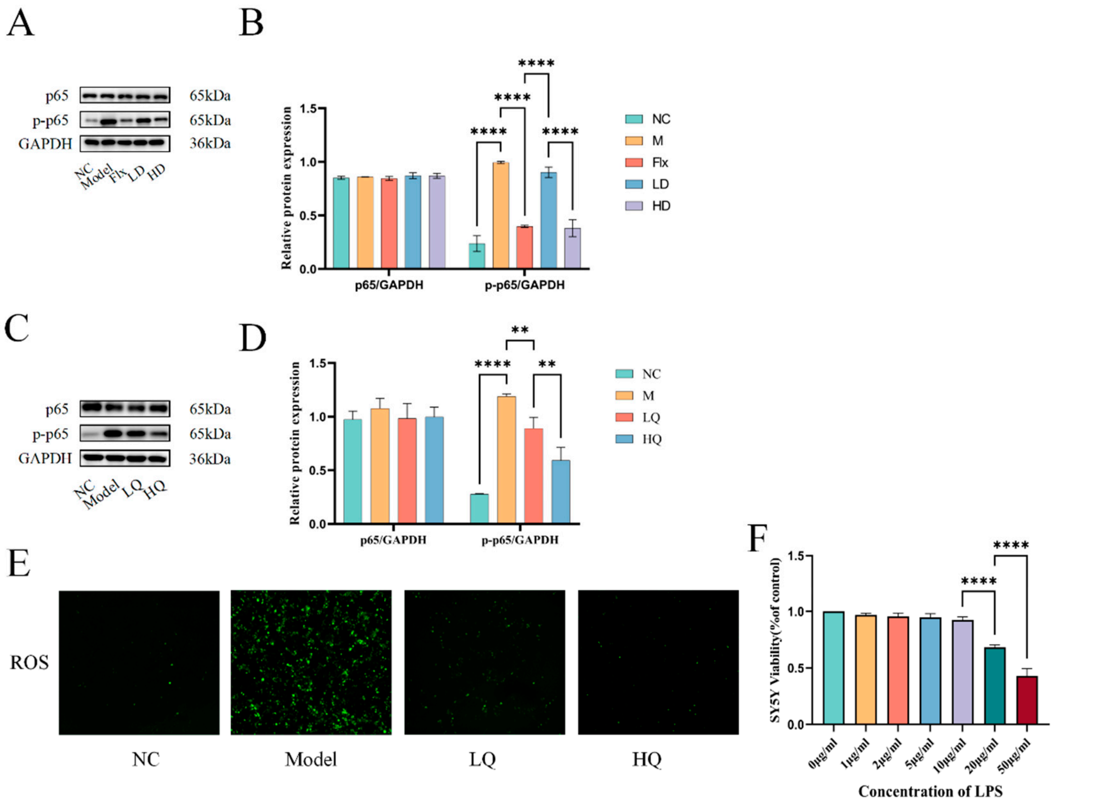

Figure S12. HCB regulated NF-κB signaling pathway and ROS (A) Representative protein bands of p65 and p-p65 in hippocampal. (B) Statistical graphs of relative protein expression of ratio of p-65/GAPDH, p-p65/GAPDH Data are presented as mean ± SEM, ****P <0.0001.(C) Representative protein bands of p65 and p-p65 in SY5Y. (D) Statistical graphs of relative protein expression of ratio of p-65/GAPDH, p-p65/GAPDH Data are presented as mean ± SEM, **P<0.01, ****P <0.0001.(E) HCB can alleviate ROS production induced by LPS at the cellular level.(F)CCK8 results for LPS.
